# Assessing the capabilities of 2D fluorescence monitoring in microtiter plates with data-driven modeling for secondary substrate limitation experiments of *Hansenula polymorpha*

**DOI:** 10.1186/s13036-023-00332-0

**Published:** 2023-02-13

**Authors:** Christoph Berg, Laura Herbst, Lisa Gremm, Nina Ihling, Olivier Paquet-Durand, Bernd Hitzmann, Jochen Büchs

**Affiliations:** 1 AVT - Aachener Verfahrenstechnik, Biochemical Engineering, RWTH Aachen University, Forckenbeckstraße 51, 52074 Aachen, Germany; 2 Department of Process Analytics & Cereal Science, Institute for Food Science and Biotechnology, University of Hohenheim, Garbenstraße 23, 70599 Stuttgart, Germany

**Keywords:** 2D fluorescence spectroscopy, Online monitoring, Multivariate data analysis, High-throughput, Microbioreactor, Microtiter plate, Secondary substrate limitation, *Hansenula polymorpha*

## Abstract

**Background:**

Non-invasive online fluorescence monitoring in high-throughput microbioreactors is a well-established method to accelerate early-stage bioprocess development. Recently, single-wavelength fluorescence monitoring in microtiter plates was extended to measurements of highly resolved 2D fluorescence spectra, by introducing charge-coupled device (CCD) detectors. Although introductory experiments demonstrated a high potential of the new monitoring technology, an assessment of the capabilities and limits for practical applications is yet to be provided.

**Results:**

In this study, three experimental sets introducing secondary substrate limitations of magnesium, potassium, and phosphate to cultivations of a GFP-expressing *H. polymorpha* strain were conducted. This increased the complexity of the spectral dynamics, which were determined by 2D fluorescence measurements. The metabolic responses upon growth limiting conditions were assessed by monitoring of the oxygen transfer rate and extensive offline sampling. Using only the spectral data, subsequently, partial least-square (PLS) regression models for the key parameters of glycerol, cell dry weight, and pH value were generated. For model calibration, spectral data of only two cultivation conditions were combined with sparse offline sampling data. Applying the models to spectral data of six cultures not used for calibration, resulted in an average relative root-mean-square error (RMSE) of prediction between 6.8 and 6.0%. Thus, while demanding only sparse offline data, the models allowed the estimation of biomass accumulation and glycerol consumption, even in the presence of more or less pronounced secondary substrate limitation.

**Conclusion:**

For the secondary substrate limitation experiments of this study, the generation of data-driven models allowed a considerable reduction in sampling efforts while also providing process information for unsampled cultures. Therefore, the practical experiments of this study strongly affirm the previously claimed advantages of 2D fluorescence spectroscopy in microtiter plates.

**Supplementary Information:**

The online version contains supplementary material available at 10.1186/s13036-023-00332-0.

## Background

Fluorophores are molecules that can undergo absorption and emission of energy upon excitation of light at a compound-specific wavelength. The radiative emission of fluorescent light occurs, when an electron of a singlet excited state returns to the ground state. During this process, energy is partially dissipated by non-radiative vibrational relaxation. Consequently, fluorescence emission is observed at an increased wavelength compared to the excitation wavelength [1]. Coenzymes such as flavin adenine dinucleotide (FAD), Nicotinamide adenine dinucleotide (NADH), or aromatic amino acids such as tryptophane and phenylalanine are naturally occurring intracellular fluorophores. Among others, their fluorescence intensity depends on the intracellular concentration or the pH value. This molecular feature has been exploited for online bioprocess monitoring for over 50 years [2–4]. Over the time, the spectroscopical setups have advanced from the measurements of single wavelength combinations to scanning measurements using filter wheels [5–7] and tunable grating-based monochromators [8–10]. Recently, by introducing charge-coupled device (CCD)-based detectors, even measurement of continuous emission spectra was enabled in stirred tank reactors [11] and microtiter plates (MTPs) [12].

With the increased amount of available spectral data, also workflows and machine learning methods had to be adapted to the needs of bioprocess engineering. Mowbray et al. classified the available algorithms into the groups of multivariate data analysis (MVDA), support vector machines, ensemble learning, artificial neural networks and Gaussian processes [13]. Of these, especially methods of MVDA, such as principal component analysis (PCA) and partial least-square (PLS) regression, have routinely been applied within the related field of chemometrics [13, 14]. PCA is a dimension reduction technique, which allows the description of high dimensional data by only a few principal components (PCs). The PCs are iteratively generated, according to the maximum variance of the residual data in an orthogonal manner [15]. Thereby, besides dimensional reduction, PCA can be applied to separate dominant and less dominant spectral signal dynamics. This additional feature has successfully been used for describing cultivation processes in more detail [16, 17]. PLS regression is a supervised machine learning technique closely related to PCA. Instead of solely relying on independent spectral data, an additional dependent response dataset, such as a compound concentration, is included to generate covariance-based regression models. Applying the regression models to external spectral data, allows the prediction of the respective response variable without the need for offline measurement [18]. Within the field of bioprocess engineering, the implementations and applications of spectroscopic setups in combination with machine learning algorithms are constantly advancing and are, thus, frequently re-evaluated [13, 19–23].

Online fluorescence monitoring in high-throughput microbioreactors allows cost-efficient, parallel screening experiments. This advantage renders the technology an invaluable tool during early-stage bioprocess development [24–26]. Successful applications have been reported for clone screening and genetic construct selection [27–31], substrate feeding [32, 33] and induction profiling [10, 34, 35]. Also, a systematic evaluation of media compositions and cultivation conditions for improved microbial growth and product formation has been demonstrated [36–39]. For the recently introduced 2D fluorescence spectroscopy in MTPs, a successful proof-of-concept was shown for the cultivation of *Escherichia coli* and *Hansenula polymorpha* [12, 40–42]. In these first studies, data-driven PLS modeling was extensively tested for the variation of carbon sources and initial cell dry weights (CDW). In another study, scattered-light spectra were generated for the co-cultivation of *Lactococcus lactis* and *Kluyveromyces marxianus.* Data-driven modeling was used to calculate the coculture composition [43]*.* Furthermore, the setup has been used for analysing autofluorescence and absorption of a pigment in *Trichoderma reesei* RUT- C30 and *Streptomyces coelicolor* A3 (2) mixed cultures [44–46].

This study aims to evaluate the capabilities of the new MTP-based monitoring system under more practical-oriented conditions. The methodology for 2D fluorescence-based PLS modeling, presented by Berg et al. [41], is applied to experimental data of higher complexity. Following the *H. polymorpha* screening experiments of Kottmeier et al. [47], three separate experiments are conducted, with each varying the supplementation of one secondary substrate of magnesium (Mg^2+^), potassium (K^+^), or phosphate (PO_4_^3−^). 2D fluorescence spectroscopy is used to monitor the dynamics of GFP and biomass accumulation, while further metabolic insights are obtained by the measurement of the oxygen transfer rate (OTR) and by extensive offline sampling. Subsequently, the spectral data of cultures of two cultivation conditions in combination with a subset of the offline data is then used to generate PLS models, before internal and external validation is conducted using data not used for calibration. Based on the results and the comparison to the study by Berg et al. [41], the capabilities and limitations of the 2D fluorescence monitoring technology in MTPs are assessed.

## Results

### Magnesium limitation experiment

**Fig. 1:**
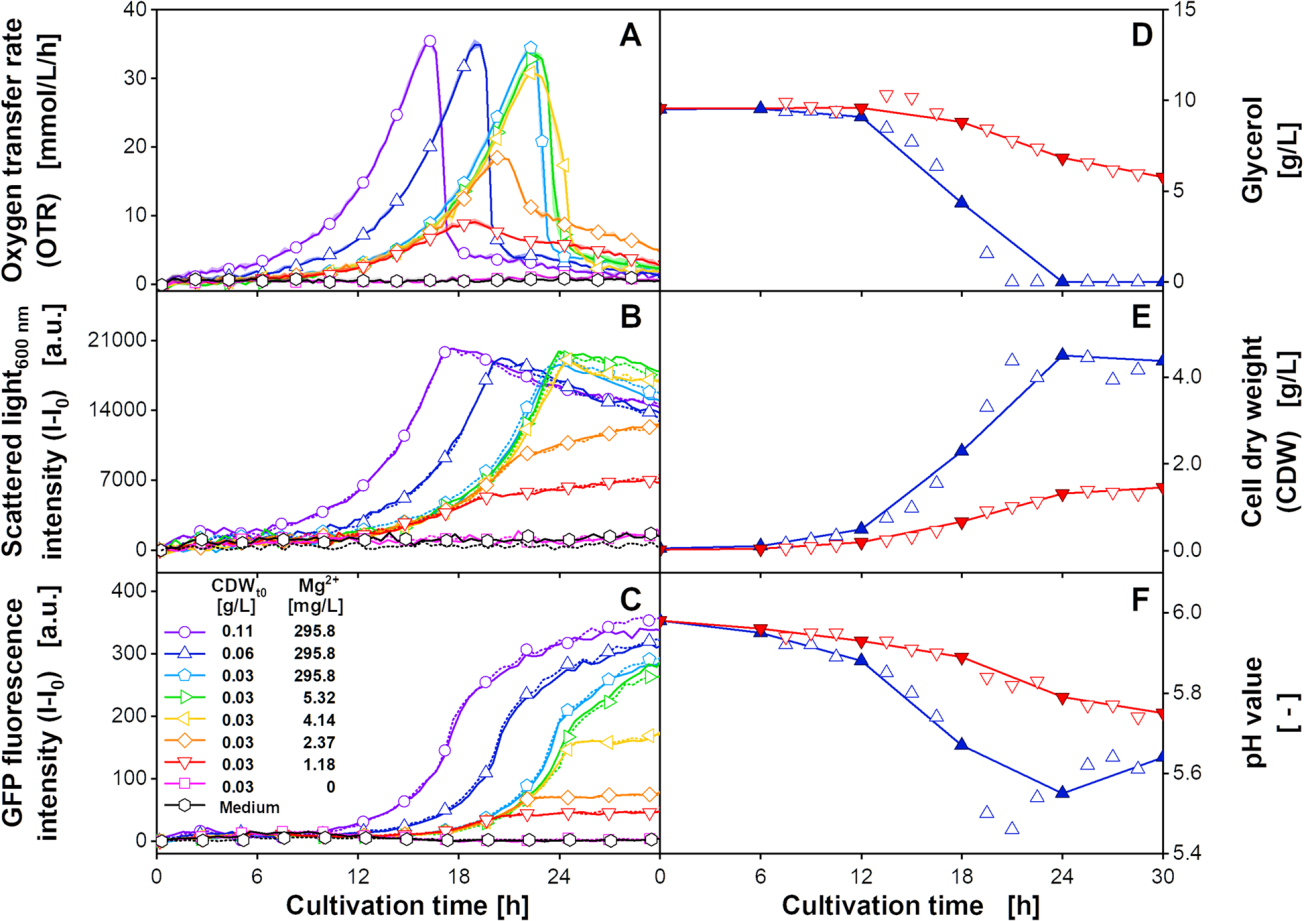
Time-resolved (**A-C**) online monitoring signals and (**D-F**) offline sample measurements of *H. polymorpha* RB11 pC9-*FMD* (P_*FMD*_-GFP) cultivations at different initial cell dry weight (CDW_t0_) and magnesium (Mg^2+^) concentrations. (**A**) The mean oxygen transfer rate (OTR) of culture replicates (*n* = 2–3, Additional file 1:Fig. S3) was determined by a μRAMOS device [48]. The low standard deviations are shown as shaded areas and indicate good reproducibility. Hollow symbols indicate every sixth data point. (**B**) Scattered light (λ_ex_ = λ_em_ = 600 nm) and (**C**) GFP fluorescence intensities (λ_ex_ = 420 nm, λ_em_ = 530 nm) were extracted from 2D spectra of duplicates (solid and dotted lines). Hollow symbols indicate every fifth data point. Values of (**D**) glycerol, (**E**) cell dry weight (CDW), and (**F**) pH value are based on singular offline measurement from parallel cultivation, taken every 1.5 h (hollow and filled symbols). Filled, linearly interpolated symbols describe a sampling interval of 6 h. Cultivation conditions: 48-well microtiter plate with round geometry, modified SYN6-MES medium, liquid volume = 800 μL, shaking diameter = 3 mm, shaking frequency = 1000 rpm, temperature = 30 °C

In the first cultivation experiment, the magnesium (Mg^2+^) supplementation of the modified SYN6-MES medium was varied between 295.8 mg/L and 0 mg/L. In addition to the initial cell dry weight (CDW_t0_) of 0.03 g/L, for the cultures with 295.8 mg/L magnesium also 0.06 g/L and 0.11 g/L were cultivated. Following the experimental layout described in Additional file 1: Fig. S1, the resulting online data of the OTR, scattered light and GFP fluorescence is shown in Fig. 1A-C. Exemplary recorded 2D spectra are shown in Additional file 1: Fig. S2. Offline sampling data for the cultures with 295.8 mg/L magnesium and a CDW_t0_ of 0.06 g/L (blue upward triangle) and 1.18 mg/L magnesium and a CDW_t0_ of 0.03 g/L (red downward triangle), taken at a 1.5 h interval, are shown as hollow symbols in Fig. 1D-F. The filled, linearly interpolated symbols describe a more realistic sampling interval of 6 h and are later used for PLS model calibration.

The OTR signals in Fig. 1A are comparable to the results of Kottmeier et al. [47], although being obtained in MTPs instead of shake flasks. The cultures with a magnesium supplementation of 295.8 mg/L and a CDW_t0_ of 0.03 g/L (light blue pentagons) showed exponential growth until 22.3 h, with a maximum OTR value of 34.5 mmol/L/h. Afterwards, the OTR dropped below 5 mmol/L/h. Both the scattered light and the GFP signals followed this exponential growth pattern. Shortly before the maximum OTR was reached, a sudden increase was observed for the GFP signal, which is connected to the partial derepression of the *FMD* promotor due to low glycerol concentrations [49, 50]. After the increase, the GFP signals flattened asymptotically. The cultures with an increased CDW_t0_ (purple circles, blue upward triangles) described an earlier exponential increase, but were otherwise similar. With decreasing the magnesium supplementation, the maximum OTR was reduced, and the final signal drop was successively replaced by a slight linear decrease. Accordingly, the scattered light signal was observed to transition from an exponential increase to a linear increase upon reaching the OTR maximum. For the GFP signal, a sudden stagnation was observed for the cultures supplemented with a magnesium supplementation of up to 4.14 mg/L (yellow leftward triangles). No considerable metabolic activity was measured for the cultures without magnesium (pink squares) and the wells containing only non-inoculated medium (black hexagons).

The offline data in Fig. 1D-F described an exponential increase of the CDW and an according glycerol and pH decrease during the unlimited growth phase. In total, around 4.1 g/L CDW were accumulated for the culture supplemented with 295.8 mg/L magnesium. The pH value decreased to a minimum of 5.48. For the culture supplemented with 1.18 mg/L magnesium, the transition from an exponential OTR increase to a linear decrease resulted in a slow linear decrease and a residual glycerol concentration of 5.5 g/L at the end of the cultivation. Here, a total of 1.9 g/L CDW was accumulated, while the pH decreased to 5.75. For the 6 h sampling interval the exponential growth pattern was described inaccurately, as especially the time of glycerol depletion and the minimum pH value were not correctly reflected.

**Fig. 2:**
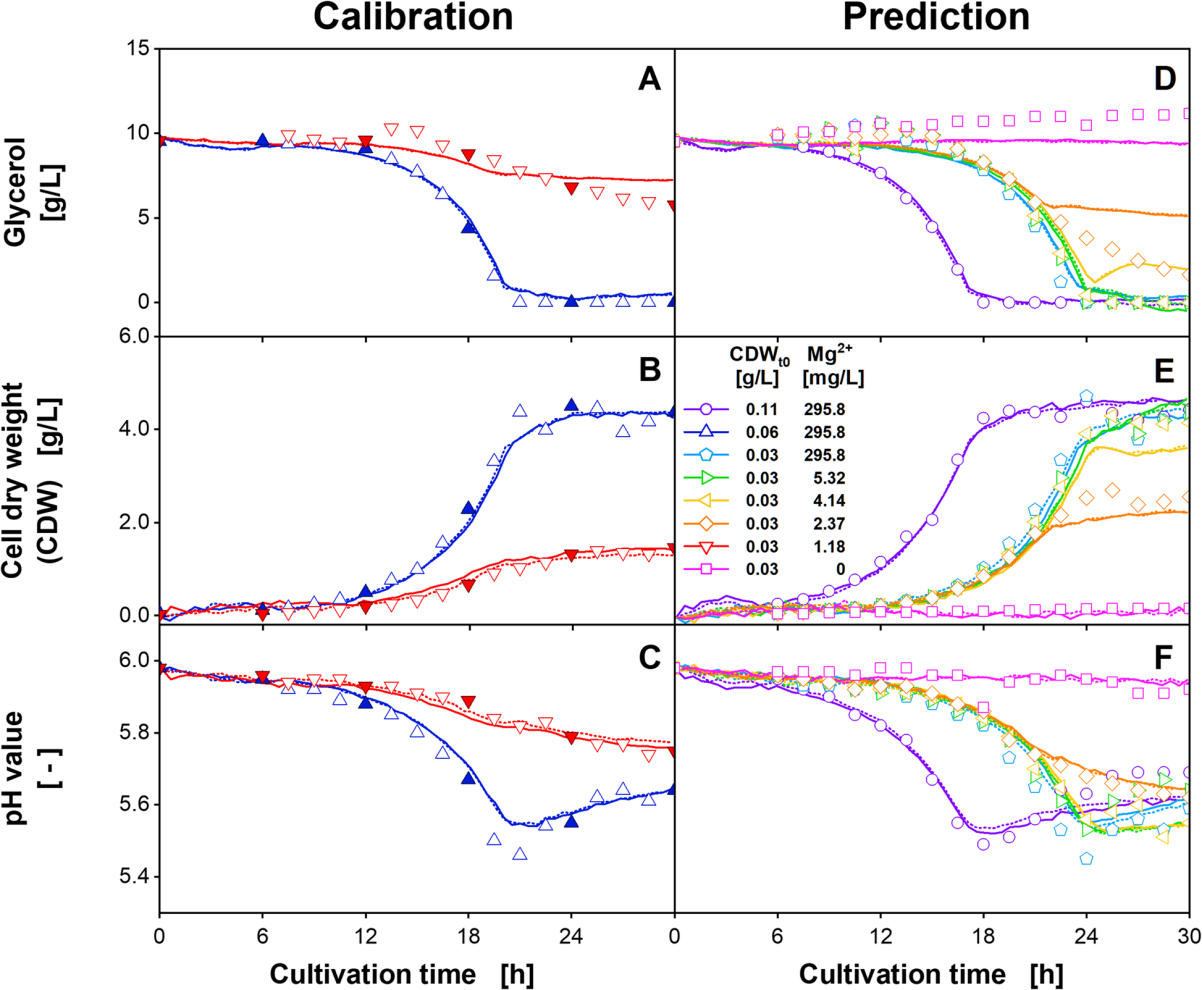
(**A-C**) Calibration and (**D-F**) prediction of the PLS models for three offline parameters for *H. polymorpha* RB11 pC9-*FMD* (P_*FMD*_-GFP) cultivations at different initial cell dry weight (CDW_t0_) and magnesium (Mg^2+^) concentrations. This figure is based on the data in Fig. 1. The PLS models were calibrated using the linearly interpolated values of the filled symbols and a total of 248 2D spectra of the two cultivation conditions shown in **A-C**. The PLS models for (**A**, **D**) glycerol, (**B**, **E**) CDW, and (**C**, **F**) pH value were generated using two, three, and two LVs, respectively. Solid and dotted lines describe the calculated parameter progression of duplicates. Hollow symbols in **A**-**F** were used for model validation only. Cultivation conditions: 48-well microtiter plate with round geometry, modified SYN6-MES medium, liquid volume = 800 μL, shaking diameter = 3 mm, shaking frequency = 1000 rpm, temperature = 30 °C

Similar to the study by Berg et al. [41], the PLS models were generated based on online spectral data of two replicates and the linearly interpolated offline values of the sparse, 6 h sampling interval of the cultures shown in Fig. 1D-F. The prediction dataset consisted of the spectral data of the remaining cultivation conditions shown in Fig. 1A-C. The OTR was not used for PLS modeling. The resulting root-mean-square error (RMSE) for different numbers of latent variables (LVs) are shown in Additional file 1: Fig. S4. The appropriate number of LVs was identified by a decreasing RMSE_Cal, sparse_, accompanied by an increase of the RMSE_Cal, full_. According to Berg et al. [41], at this point, the model starts to overfit the sparse data, instead of representing the inherent biology-based spectral dynamics. Following this methodology, the PLS models were generated using two LVs for the glycerol concentration and the CDW and three LVs for the pH value. The calculated offline parameter progressions for the calibration and the prediction dataset are shown in Fig. 2.

In Fig. 2A, the glycerol values calculated for the cultures with 295.8 mg/L magnesium (blue upward triangles) agreed well with the exponential decrease, described by the offline values of the short sampling interval. The lowest glycerol concentration is calculated at 0.15 g/L and coincided with the depletion of glycerol depletion according to the sparse sampling interval. For the cultures with 1.18 mg/L magnesium (red downward triangles), the initial non-linear glycerol decrease was calculated appropriately. However, according to the PLS model, glycerol consumption ceases after 19 h. This led to a final difference of 1.2 g/L between the calculated and the measured values, which represented the time point of the onset of limitation and the stagnating GFP signal (Fig. 1C). For the prediction dataset (Fig. 2D), good predictability of the glycerol concentration was observed during exponential growth, while under magnesium-limiting conditions, the glycerol consumption was underestimated. As a result, especially for the cultures supplemented with 2.37 mg/L (orange diamonds) and 4.14 mg/L magnesium (yellow leftward triangles), the calculated values considerably exceeded the measured values. In total, an RMSE_Cal, full_ of 0.57 g/L was calculated, while for the prediction dataset, an RMSE_Pred, full_ of 0.94 g/L resulted. Relative to the measured glycerol range of 11.98 g/L, this accounts for 5.1% for the calibration dataset and 8.5% for the prediction dataset (Additional file 2: Table S1).

The results for the PLS model of the CDW are shown in Fig. 2B and E. The CDW was accurately calculated for both cultivation conditions of the calibration dataset. For the prediction dataset, the calculated values agreed well with the measured values during the exponential growth. However, especially for the increasingly limited cultures, the PLS model described a premature growth stagnation. This stagnation led to the final CDW values being underestimated by as much as 0.5 g/L for the cultures with 4.14 mg/L magnesium. In conclusion, an RMSE_Cal, full_ of 0.18 g/L (3.8%), and an RMSE_Pred, full_ of 0.3 g/L (6.5%) were determined (Additional file 2: Table S1).

The PLS model of the pH value is shown in Fig. 2C and F. The model accurately reflected the initial pH decrease and the subsequent increase for the calibration cultures with 295.8 mg/L magnesium. However, the minimum value measured at 5.46 was calculated to be 5.55, which represented the lowest value of the 6 h sampling dataset used for calibration. For the culture supplemented with 1.18 mg/L magnesium, the PLS model accurately described the pH value throughout the cultivation time. Transferring the model to the prediction dataset resulted in comparable observations. While the pH values of the strongly limited cultures were described accurately, deviations increased for the cultures of higher magnesium. This was especially observed for the time after the metabolic activity ended. A systematic overestimation of the pH values was observed for the cultures with a CDW_t0_ of 0.03 g/L of 295.8 mg/L magnesium (light blue pentagons). In summary, an RMSE_Cal, full_ of 0.025 (4.9%) was obtained, while for the RMSE_Pred, full_ a value of 0.032 (6.0%) resulted.

In conclusion, despite the changes in the scattered light and fluorescence dynamics, the resulting PLS models provide a reasonable overall accuracy. Although for individual cultivation conditions the predictive performance showed shortcomings of the models, with a relative RMSE below 10%, the acceptance criterium for a transferable PLS model described by Yousefi-Darani et al. [51] is met.

### Potassium limitation experiment

In the second experiment, the potassium (K^+^) supplementation to the modified SYN6-MES medium was varied. As for the previous experiment, the calculated RMSE included three very similar cultivation conditions, the variation of the CDW_t0_ was omitted. Thereby, also the number of cultures grown under limited conditions could be increased. In total, eight cultivation conditions with potassium concentrations between 2017.3 mg/L and 20.2 mg/L were investigated.

With decreasing potassium concentration, the online spectral data in Additional file 1: Fig. S5A and B described a transition into a decelerated linear increase for both the scattered light and the GFP intensity. This resulted in the maximum intensities being decreased by more than for the cultures with the lowest potassium concentration of 20.2 mg/L when compared to the fully supplemented cultures. Accordingly, also the offline data in Additional file 1: Fig. S5C-E described a reduced, linear growth and consumption during the limitation phase. Comparable to the previous experiment, the linear interpolation of the sparse sampling interval did not accurately describe the non-linear growth pattern and missed the exact time of glycerol depletion, as well as the correct minimum pH value.

**Fig. 3:**
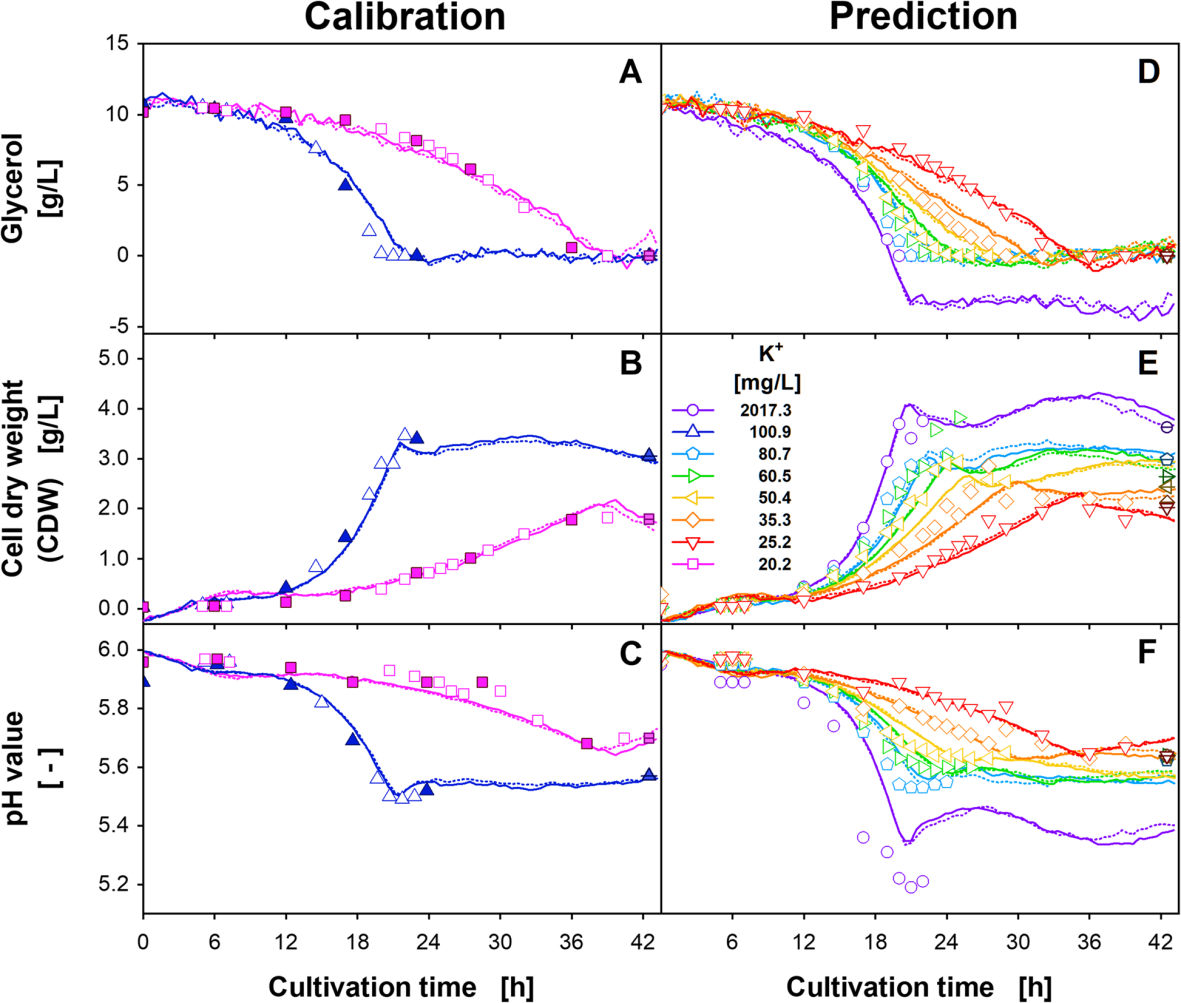
**(A-C)** Calibration and (**D-F**) prediction of the PLS models for three offline parameters of *H. polymorpha* RB11 pC9-*FMD* (P_*FMD*_-GFP) cultivations at different potassium (K^+^) concentrations. This figure is based on the data from Additional file 1: Fig. S5. The PLS models were calibrated with the linearly interpolated values of the filled symbols and a total of 356 2D spectra of the two cultivation conditions shown in **A**-**C**. The PLS models for (**A**, **D**) glycerol, (**B**, **E**) CDW, and (**C**, **F**) pH value were generated using four, three, and three LVs. Solid and dotted lines describe the calculated parameter progression of duplicates. The hollow symbols were used for model validation only. The crossed symbols describe the value for the last offline sample taken after 54 h. Cultivation conditions: 48-well microtiter plate with round geometry, modified SYN6-MES medium, liquid volume = 800 μL, shaking diameter = 3 mm, shaking frequency = 1000 rpm, temperature = 30 °C

Similar to the previous experiment, the PLS model was generated based on the spectral online and the sparse offline sampling data of the cultures with the second highest (100.9 mg/L potassium, blue upward triangles) and the lowest (20.2 mg/L potassium, pink squares) potassium supplementation. The prediction dataset consisted of duplicates of the remaining online monitored cultivation conditions, shown in Additional file 1: Fig. S5A-B. The RMSEs resulting from the variation of LVs are shown in Additional file 1: Fig. S6. The RMSE_Cal, sparse_ and RMSE_Cal, full_ were of the same magnitude, as observed for the magnesium limitation experiment. Similarly, a continuous decrease of the calculated values was observed for increasing LVs. An overall reduced RMSE was observed, when excluding the cultures with the highest potassium concentration from the prediction dataset (RMSE_Pred, full, − 100%,_ blue diamonds). Following the previously described criteria for the final PLS models of the CDW and the pH value, three LVs were used, while for the glycerol concentration, four LVs were chosen. The resulting trajectories, calculated by the PLS models, are shown in Fig. 3.

In Fig. 3A and D, the calculated glycerol values showed a tendency towards the values of the interpolated sparse sampling interval. Consequently, for both the calibration and the prediction dataset, the glycerol progressions were described more accurately for lower potassium concentrations. For higher potassium supplementation, the glycerol consumption rates were underestimated, leading to the calculated glycerol depletion to occur later than determined by the measurements. For the culture supplemented with 2017.3 mg/L potassium (purple circles), the glycerol concentration was calculated to a value of − 3.5 g/L after 21 h. Here, a close connection to the increased scattered light (Additional file 1: Fig. S5A) can be assumed. In total, the determined RMSE_Cal, full_ is 0.51 g/L (4.7%), while for the prediction dataset, the RMSE_Pred, full_ was calculated to 2.05 g/L (18.7%). Excluding the cultures with the highest potassium concentration resulted in a considerably reduced RMSE_Pred, full, − 100%_ of 0.57 g/L (5.2%).

As for the glycerol concentration, also for the PLS model of the CDW, shown in Fig. 3B and E, higher inaccuracies were observed for higher potassium concentrations resulting from an underestimated growth rate. Only for the cultures with 2017.3 mg/L potassium, the calculated and the measured offline values were again in very good alignment during the exponential growth. However, after the exponential growth was terminated (between 18 h and 42 h) non-plausible CDW fluctuations with deviations of up to 0.45 g/L were calculated. As a result, an RMSE_Cal, full_ of 0.15 g/L (4.0%) was calculated, while the RMSE_Pred, full_ was determined to 0.55 g/L (14.6%). The RMSE_Pred, full, − 100%_ was reduced to 0.29 g/L (8.4%) as the described fluctuations were omitted.

The calculated values for the pH value of the calibration dataset in Fig. 3C show a tendency of underestimation for the low potassium supplementation and an overestimation for the high potassium supplementation. When applied to the prediction dataset (Fig. 3F), the PLS model accurately predicted the pH values for the cultures with potassium concentrations of up to 35.3 mg/L (orange diamonds). However, for higher potassium concentrations, the accuracy decreased. Considerable shortcomings were observed for the cultures of 2017.3 mg/L potassium, for which the measured minimal pH value was missed by 0.14. In total, the RMSE_Cal, full_ of the PLS model was calculated to 0.034 (4.3%), while the RMSE_Pred, full_ was calculated to 0.054 (6.9%). When excluding the cultures with the highest potassium concentration, the RMSE_Pred, full, − 100%_ was reduced to 0.032 (6.5%). While this represents only a limited reduction of the percentage value, compared to the RMSE_Pred, full_, the absolute RMSE_Pred, full, − 100%_ is nearly reduced by half.

In conclusion, the potassium experiment showed lower PLS modeling performance than the magnesium variation experiment. This partially resulted from the chosen cultivation conditions and the reduced similarity between the calibration and prediction dataset.

### Phosphate limitation experiment

In the third experiment, the supplementation of phosphate (PO_4_^3−^) was varied between 0 mg/L and 697.9 mg/L. The resulting online and offline values are shown in Additional file 1: Fig. S7. The OTR signals indicated the onset of a limitation for phosphate concentrations of 139.6 mg/L (light blue pentagons) or less. The limitation led to a decreased maximum of the OTR, followed by a plateau of variable length. Concurrently, the scattered light transitioned to a linear increase, while the GFP signal stagnated. Upon the final decrease of the OTR, an additional final increase of the GFP intensity was observed, the extend of which decreased with decreasing phosphate supplementation. Noteworthy, for all phosphate concentrations, the scattered light signals reached comparable maximum values. For the cultures without phosphate (pink squares) and the non-inoculated medium (black hexagons), no considerable signal increase was observed.

Similar to the scattered light, the offline data in Additional file 1: Fig. S7D-F indicated a comparable CDW of 3.2 g/L CDW for both sampled conditions, despite a slower growth of the limited culture. In contrast, the higher phosphate supplementation led to a minimum pH value of 5.44, whereas for the lower supplementation a pH minimum of 5.8 was observed. Again, the linear interpolation of the sparse sampling interval introduced inaccuracies for the timing of the depletion of glycerol, the maximum CDW, and the minimum pH value for the cultures with the higher phosphate supplementation. For the culture of lower phosphate supplementation, the trajectory was appropriately described.

**Fig. 4:**
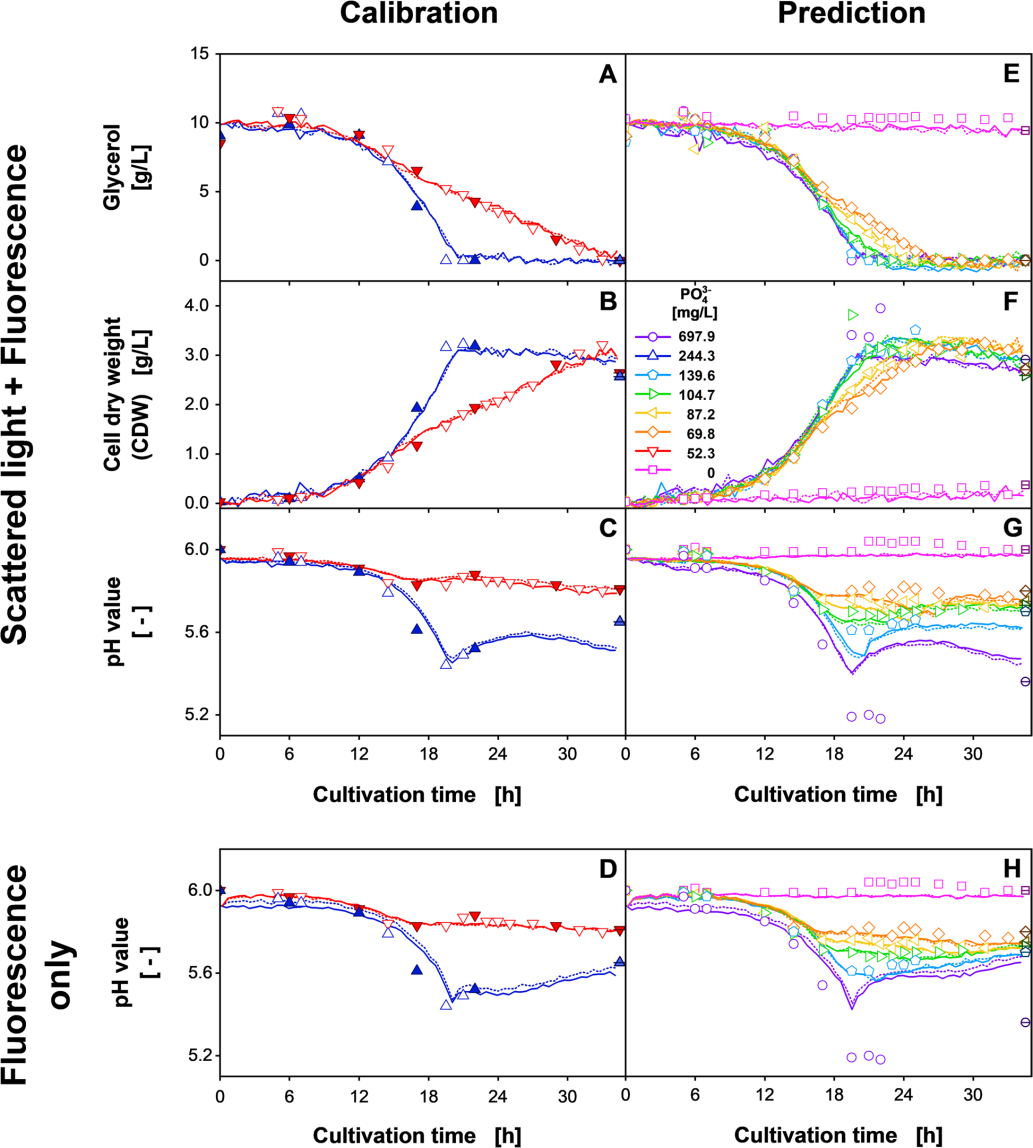
(**A-D**) Calibration and (**E-H**) prediction of the PLS model for three offline parameters, based on 2D spectra (**A-C**, **E-G**) including and (**D**, **H**) excluding the scattered light of *H. polymorpha* RB11 pC9-*FMD* (P_*FMD*_-GFP) cultivations at eight different phosphate (PO_4_^3−^) concentrations. This figure is based on the data in Additional file 1: Fig. S7. The PLS models were calibrated with the linearly interpolated values of the filled symbols and a total of 288 2D spectra from the two cultivation conditions shown in **A-C**. The models in Fig. 4D and H were generated including only the fluorescence intensities. The PLS models for (**A**, **E**) glycerol, (**B**, **F**) CDW, and (**C**, **D**, **G**, **H**) pH value were generated using five, five, and three latent variables, respectively. Solid and dotted lines describe the predicted parameter progression of duplicates. Crossed symbols describe the value for the last offline sample taken after 44 h. Cultivation conditions: 48-well microtiter plate with round geometry, modified SYN6-MES medium, liquid volume = 800 μL, shaking diameter = 3 mm, shaking frequency = 1000 rpm, temperature = 30 °C

The PLS models were generated based on the data of the first 34.5 h of cultivation. The calibration dataset included the cultivation conditions shown in Additional file 1: Fig. S7D-F, while the prediction dataset consisted of the remaining inoculated cultures. With increasing LVs, the RMSE_Cal, full_, and the RMSE_Cal, sparse_ of the PLS models continuously decreased for all offline parameters as shown in Additional file 1: Fig. S9A-C. The two calibration RMSEs of the glycerol concentration decreased in parallel, while for the CDW, the RMSE_Cal, full_ surpasses the RMSE_Cal, sparse_ for five LVs. For the pH value, the two values were nearly identical for all eight LVs. For the prediction dataset, the glycerol and the CDW concentration showed a constant positive offset of the RMSE_Pred, full_ to the RMSEs of the calibration datasets. Only for the pH value, a valley-shaped progression with a minimum of three LVs was observed. For this parameter, excluding the cultures of the highest phosphate supplementation resulted in a minimum RMSE_Pred, full, − 100%,_ which was comparable to the RMSEs of the calibration datasets. In conclusion, the criterium for choosing the number LVs [41] did only apply for the CDW and led to five LVs. However, for the glycerol concentration and the pH value the criterium did not apply. Thus, five and three LVs were chosen subjectively, as these represented the minimum values of the RMSE_Pred, full, − 100%_. The resulting PLS models are shown in Fig. 4.

Comparable to the previous experiment, the PLS model for glycerol in Fig. 4A and E showed a tendency towards linear glycerol consumption for both the calibration and the prediction dataset. Consequently, the cultures with lower phosphate concentrations were predicted more accurately, while the exponentially grown cultures with phosphate supplementation of at least 244.3 mg/L showed slight deviations from the measured values. As a result, the RMSE_Cal, full_ was determined to 0.58 g/L (5.2%). With a RMSE_Pred, full_ of 0.61 g/L (5.6%) and a RMSE_Pred, full, − 100%_, of 0.59 g/L (5.4%), very comparable values were achieved for the transfer to the prediction dataset.

Also, for the calculated CDW in Fig. 4B and F, a better predictability for the cultures with lower phosphate supplementation was observed. However, when transferring the model to the prediction dataset, the noise of the calculated values significantly increased, while the reproducibility of the replicates decreased. Additional, notable deviations from the measured offline values were observable for the cultures with 104.7 mg/L (green rightward triangles) and 697.9 mg/L phosphate (purple circles) between 19 h and 21 h of cultivation. While for the lower phosphate supplementation, a singular offline measurement error is conceivable, for the higher supplementation, the maximum calculated CDW of 3.0 g/L underestimated multiple offline measurements, ranging between 3.4 g/L and 3.9 g/L. Therefore, here, an inaccurate PLS model is more likely. In total, the RMSE_Cal, full_ was calculated to be 0.12 g/L (3.0%), while the observed deviations of the prediction dataset resulted in an RMSE_Pred, full_ of 0.26 g/L (6.6%). Excluding the cultures with the highest phosphate supplementation resulted in a reduced RMSE_Pred, full, − 100%_, of 0.16 g/L (4.3%).

The PLS modeling results for the pH value, shown in Fig. 4C, are in accordance with the glycerol concentration and the CDW. Again, the calculated values for the limited cultures described the PLS models more adequately compared to the cultures with high phosphate supplementation, which were systematically overestimated between 12 h and 19.5 h of cultivation. Nevertheless, the model correctly reflected the minimum pH value of 5.44, which was not part of the calibration dataset. After the glycerol is consumed, the model eventually calculated a second, non-apparent decrease in the pH value for the high phosphate supplementation. Transferring the PLS model to the prediction dataset resulted in more systematic deviations, as shown in Fig. 4G. The PLS model appropriately reflected the decline of the pH value during the initial exponential growth phase. However, especially for the cultures with 87.2 mg/L (yellow leftward triangles) and 69.8 mg/L phosphate (orange diamonds), the pH values were calculated to further decrease until the end of the metabolic activity, despite being measured to be constant. After the metabolic activity had ceased, the calculated pH value increased to values approximating the measured values. For the cultures supplemented with 697.9 mg/L phosphate, the calculated progression of the pH value was qualitatively comparable to the cultures supplemented with 244.3 mg/L phosphate (blue upward triangles). However, the minimum calculated pH value was 5.4 instead of the measured minimum value of 5.19. In conclusion, the RMSE_Cal, full_ was calculated to be 0.038 (3.8%), whereas the described inaccuracies of the prediction dataset resulted in an RMSE_Pred, full_ of 0.085 (8.5%) and a considerably reduced RMSE_Pred, full, − 100%_ of 0.04 (5.4%).

### Exclusion of the scattered light

In the phosphate limitation experiment, while the calibration dataset was described accurately, systematic inaccuracies were observed when transferring the PLS models to the prediction dataset. Therefore, overfitting of the calibration data in general and the spectral data in particular can be suggested. One way to investigate this hypothesis is to modify the selection of included spectral data. To exemplarily demonstrate the impact of the spectral input data, additional PLS models were generated using only the fluorescence of the 2D spectra, as shown in Additional file 1: Fig. S10.

The RMSEs for a variable number of LVs are shown in Additional file 1: Fig. S9D-F. While for a low number of LVs, the RMSEs of the calibration datasets were higher than for the PLS models including the scattered light, with increasing LVs, the RMSEs of the two models were more comparable. The same holds true for the RMSE_Pred, full_ and RMSE_Pred, full, − 100%_ of the PLS models for the glycerol concentration and the CDW. Only for the pH value, excluding the scattered light resulted in an additional reduction of the RMSE_Pred, full, − 100%_. The improved PLS modeling performance for three LVs is visualized in Fig. 4D and H.

For the cultures supplemented with 52.3 mg/L phosphate, a more constant trajectory was calculated, whereas for the culture with 244.3 mg/L phosphate, the previously observed second pH increase was no longer exhibited in the new model. For the prediction dataset in Fig. 4H, the new PLS model resulted in a considerably better alignment for the later phase of cultivation. Firstly, the systematic overestimation of the pH decrease for the strongly limited cultures was reduced. Further, also the asymptotic behavior of the pH value observed for the cultures with 139.6 mg/L phosphate after metabolic activity was terminated was described more correctly by the new model. Nevertheless, also the new model did not predict the minimum value of 5.19 for the cultures with the 697.9 mg/L phosphate correctly. In total, the PLS model including only fluorescence intensities resulted in an RMSE_Cal, full_ of 0.04 (4.0%), an RMSE_Pred, full_ of 0.106 (10.6%) and an RMSE_Pred, full, − 100%_ of 0.034 (4.6%).

**Fig. 5:**
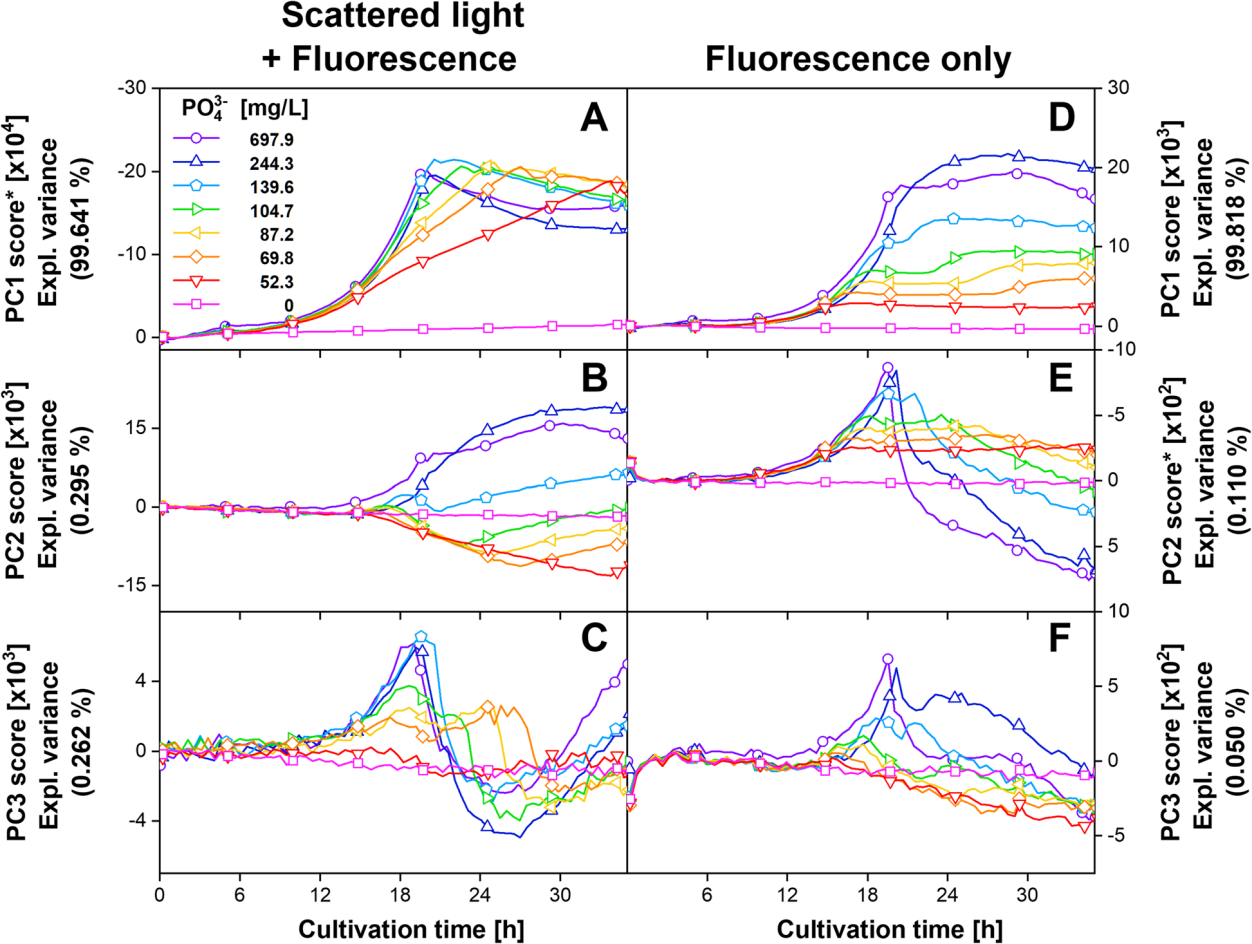
Scores of first to third principal component (PC1-PC3) over cultivation time, based on (**A-C**) 2D spectra including scattered light and fluorescence, as well as based on (**D-F**) only fluorescence intensities for the cultivation of *H. polymorpha* RB11 pC9-*FMD* (P_*FMD*_-GFP) at different phosphate (PO_4_^3−^) concentrations. The explained variance for each PC is shown in brackets. For clarity, only data of one replicate per cultivation condition is shown. Only every 10th datapoint is indicated by a symbol. Asterisks in Fig. 5A and E indicate reversed Y-axis direction used for clarity. Spectroscopic measurement settings: excitation wavelength range = 280 nm – 700 nm (step size = 10 nm), emission wavelength range = 278 nm – 720 nm (step size = 0.45 nm), integration time = 30 ms. Cultivation conditions: 48-well microtiter plate with round geometry, modified SYN6-MES medium, liquid volume = 800 μL, shaking diameter = 3 mm, shaking frequency = 1000 rpm, temperature = 30 °C

As described before, this noticeable systematic improvement of the prediction resulted from a change in the dominating spectral dynamics due to the exclusion of the scattered light. By applying PCA this change can be visualized, as shown in Fig. 5.

The scores of the first principal component (PC1) for the dataset including the scattered light accounted for an explained variance of 99.64% and resembled the scattered light intensities shown in Additional file 1: Fig. S7B. An even higher explained variance of 99.82% was achieved for the PC1 scores of the dataset including only the fluorescence intensities, for which the progression was qualitatively comparable to the GFP fluorescence shown in Additional file 1: Fig. S7C. With increasing PCs, the remaining variance is successively described. However also, the noise considerably increased, which is in good accordance with the literature [52]. For the PC2 scores of the dataset excluding the scattered light, the observed plateau strongly resembled the progression of the pH value. As it was not observed in any of the scores of the dataset including the scattered light, a connection to the improved PLS model is conceivable. The reason why the predictive performance of the model including the full dataset is lower lies in the covariance-based algorithm of the PLS regression. Thereby, although the scattered light may not be optimal for describing the pH value, it comprises a very large amount of the spectral variance and is thus also included in the model. This effect may be even increased for increasing LVs. However, by excluding the scattered light, these deteriorating online signals are no longer considered for modeling. Instead, signal dynamics for PLS model generation originate only from the inherently pH-correlating fluorescence intensities.

### Comparison of PLS models

**Fig. 6:**
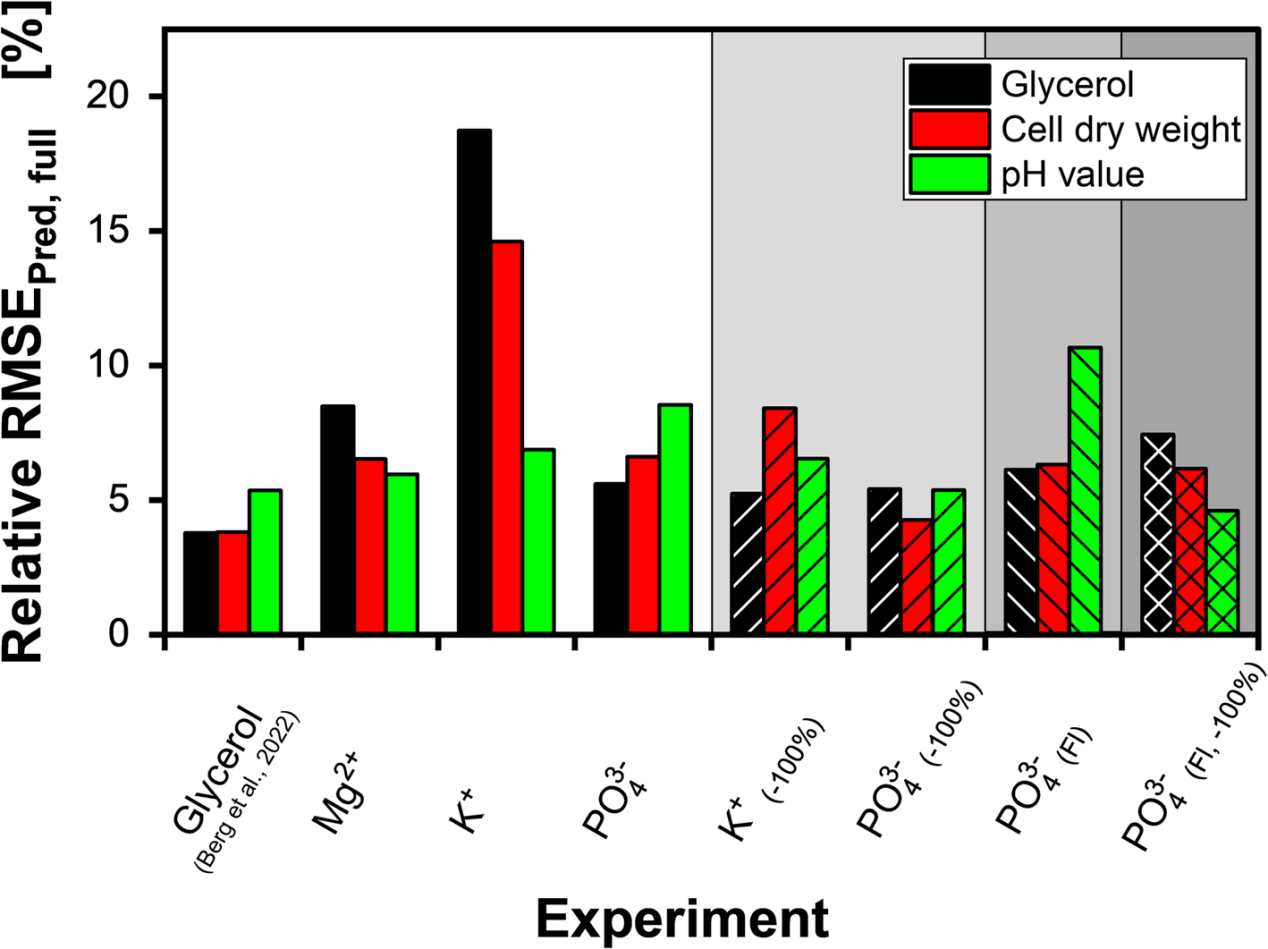
Comparison of the relative RMSE_Pred, full_ for glycerol, CDW, and pH value for the PLS models generated in Berg et al. [41] and this study. Backward diagonal hatched columns describe RMSE_Pred, full_, based on the complete prediction dataset, except the culture holding the initial concentration of the respective secondary substrate according to Jeude et al. [53] (− 100%). Forward-hatched columns describe the RMSE_Pred, full_, calculated for spectral online datasets including only the fluorescence intensities (Fl). For the diagonal cross-hatched columns, additionally, the cultures with the highest initial concentration were excluded. The individual values are given in Additional file 2: Table S1

In both, this and the previous study by Berg et al. [41], PLS regression models were generated using the same monitoring hardware and biological system. However, while the previous paper described a simple glycerol concentration variation study, in this study, more complex systems of secondary substrate limitations were investigated. As the workflow for generating the PLS models was identical, the results can directly be compared, to estimate the robustness of the methodology. In Fig. 6, a summary of the relative RMSE_Pred, full_ (plain columns) and RMSE_Pred, full, − 100%_ (backward diagonal hatched columns) is given. Additionally, also for the PLS models including only the fluorescence intensities (Fl), the RMSE_Pred, full, − 100%_ (forward diagonal hatched columns) and the respective as RMSE_Pred, full, − 100%_ (cross-hatched columns) are shown. An analogous visualisation of the absolute values is shown in Additional file 1: Fig. S11.

The glycerol-variation experiment of the previous study resulted in low relative RMSE_Pred, full,_ with values between 3.5 and 5.3%. In contrast, for all limitation experiments of this study, values above 5% were obtained. The highest relative RMSE_Pred, full_ of up to 18.7% was calculated for the glycerol concentration of the potassium limitation experiment (K^+^). A reduction by more than 10% was obtained when excluding the cultures with the highest potassium supplementation (K^+^_(− 100%)_). For the phosphate variation experiment, this exclusion procedure resulted in an RMSE_Pred, full, − 100%_ between 5.4 and 4.3%. The additional in-silico exclusion of the scattered light (PO_4_^3−^_(Fl)_) further reduced the RMSE_Pred, full, − 100%_ for the pH value from 5.4 to 4.6%. However, no considerable change in the RMSE was observed for the CDW, while for the glycerol concentration, the RMSE_Pred, full, − 100%_ even increased.

In conclusion, depending on the offline parameter, the average relative RMSE_Pred, full_ for the PLS models generated from the full 2D spectra ranged between 7.1 and 11.0%. A reduction to values between 6.0 and 6.4% was achieved for the exclusion of the cultures with the highest supplementation. Although these values still represented a considerable increase compared to the glycerol variation experiment of Berg et al. [41], with relative values below 10%, the PLS modeling results of the presented study can be considered a success. In fact, the obtained relative RMSEs are well comparable to other studies using fluorescence spectroscopic PLS modeling in stirred tank batch reactors. For example, relative values between 3.8 and 9.1% have been reported for the prediction of singular carbon sources and between 3.0 and 6.8% for the CDW [16, 54–56]. However, due to the reduced experimental throughput in stirred tank reactors, in these studies, only a limited number of different cultivation conditions was used for external validation. In conclusion, this further supports the high potential of the 2D fluorescence online monitoring technology in MTPs.

In addition to the comparison with other studies, the PLS model performance can also be compared to the respective conventional determination method of each offline parameter (i.e., HPLC, gravimetry, pH-electrode). For the glycerol concentration, the determined relative RMSE of more than 5% is considerably higher than the standard deviation of the implemented HPLC method, which was below 0.2% (data not shown). In contrast, for the CDW measurements, the determined relative RMSE is in good agreement with the reported standard deviations between 0.9 to 7% [57]. Finally, for the pH value, the absolute RMSE of around 0.05 represents the higher end of the tolerance of a single pH measurement. However, finally, it has to be stated, that the financial and personnel efforts for generating a comparable amount of offline data by manual measurements is beyond any feasibility. From this perspective, the PLS models based on 2D fluorescence spectroscopy outperform any of the conventional measurement methods.

## Discussion

Secondary substrate limitations induce metabolic reactions, which can positively impact the ratio between biomass accumulation and product formation (i.e., GFP) [37, 47]. However, in this study, the changed spectral dynamics resulted in a reduced PLS model performance compared to the previous glycerol variation study [41]. The reason for this can be found in the difference between a primary (carbon source like, i.e. glycerol) and secondary substrate (i.e., Mg^2+^, K^+^, PO_4_^3−^) limitation. For the previous study, both the biomass and the product formation ceased as soon as the glycerol concentration was depleted. This resulted in a constant relation between the two parameters and, thus, in the established PLS model to remain valid. In contrast, under secondary substrate limitation, the carbon source remained available, enabling further metabolic activity with a yet changed metabolism. With the occurring changes in the spectral dynamics and the relation between biomass and product formation, the PLS models reached the performance limits of the regression algorithm. Interestingly, for the experiments of this study, no systematic improved performance for either the limited or unlimited cultivation conditions was observed. For example, for the magnesium limitation experiment, the glycerol concentration (Fig. 2A, D) was well described for the unlimited conditions. In contrast, in the potassium limitation experiment, the PLS model of the pH value (Fig. 4C, G) shows better performance for the limited cultures. Another limitation in prediction performance was found for the fully supplemented cultures of the potassium and phosphate limitation experiment. These conditions were not covered by the calibration dataset and, thus, represented an extrapolation of the PLS model, for which the performance of the regression method is known to be low [58]. Consequently, these conditions increased the RMSE above the threshold of 10% and thus rendered the models not acceptable, according to Yousefi-Darani et al. [51]. In a subsequent data evaluation study, it should therefore be reevaluated, if a changed composition of the calibration dataset can improve the PLS modeling performance.

Besides the impact of the changing signal dynamics on the PLS modeling outcome, also the applied PLS methodology, in general, and the validation approach, in particular, should be evaluated for assessing the capabilities of the online monitoring system. An inaccurate linear interpolation of the non-linear progression of the offline parameters was used to purposely generate underperforming calibration models. However, by comparing the calibration results to the more accurate representation in form of the short sampling interval, the inaccuracies can be identified and, thus, be used for internal validation. In the previous study, this discrepancy was used to identify overfitting and as a guide for choosing the appropriate number of LVs [41]. An objective methodology for choosing the number of LVs is of high importance, as overfitted models likely include non-biogenic dynamics, such as random noise and usually lead to poorly transferable models. In practice, the previous study suggested that overfitting can be identified by an increasing RMSE_Cal,full_ and a decreasing RMSE_Cal,sparse_. In the present study, this trend was only observed for a few models, such as the CDW concentration of the magnesium (Additional file 1: Fig. S4B) or the phosphate variation experiment (Additional file 1: Fig. S9B). Presumably, the trajectories of the short and the sparse sampling interval were too similar for the limited cultures. Although, thereby, the general applicability for other experimental layouts was shown, the results of this study also point out that additional efforts to increase the robustness of the method are necessary. Thus, to generate more diverging offline trajectories, an additional in silico resampling step can be suggested. Mechanistic growth models could be used to first interpolate raw offline values before a linear interpolation is generated of selected time points. Here, especially for the secondary substrate limitation experiments, the results from PCA can be used to provide further information on the apparent growth [59, 60] and, thus, facilitate mechanistic modeling.

Another subject to elaborate on in future investigations is the chosen regression method. Although this study further supports the reported strength of the PLS regression algorithm for the inherent multicollinearity of the 2D fluorescence spectra [61], the rudimentary exclusion of the scattered light hinted at the further potential for improvement. Following the literature, the implementation of different wavelength selection algorithms can be suggested [60, 62, 63]. For example, iterative wavelength selection methods such as interval PLS [64], recursive weighted PLS [65] or optimisation-oriented methods such as ant colony optimisation [66] and genetic algorithms [67] could be used. Moreover, non-linear machine learning regression methods such as support vector machines or artificial neural networks should be considered. These advanced algorithms can possibly overcome the limitations of the linear character of the PLS regression and may even improve the prediction performance for extrapolated cultivation conditions. Finally, also the establishment of more general models should be tested. For example, a common regression model for the pH value of all three experiments of this study could be established. However, comparable to the recent study on Raman spectroscopy by Yousefi-Darani et al. [51], this demands additional workflows for spectral alignment.

Finally, the PLS modeling results should be evaluated in terms of a potential reduction in sampling efforts. As described in Additional file 1: Fig. S1, three offline MTPs were used to provide offline sampling data for model calibration, as well as internal and external validation. This extensive data allowed an estimation of the robustness of the PLS models, which was found to be decreased, in comparison to the previous glycerol concentration variation study [41]. In case the above-mentioned approaches (e.g., wavelength selection) help to improve the modeling performance for the more systematic inaccuracies, the internal and external validation could be reduced to a minimum. Comparable to the previous study, for the current study, this would allow a reduction to only one MTP for both monitoring and sampling. Nevertheless, for more complex screening layouts (e.g., inductor concentration variation screenings), the demand for monitored cultivation conditions and offline samples may even be higher than the provided 48 wells of the current system. Therefore, a scale-out to parallel MTPs monitored within the same cultivation system is desirable. For this, a first proof of concept for a monochromator-based spectroscopic setup has already been demonstrated [10].

## Conclusion

In the presented study, 2D fluorescence spectroscopy was used for online monitoring of secondary substrate-limited *H. polymorpha* cultures in MTPs. In combination with online monitoring of the OTR and extensive offline sampling, the impact of the limitations on the spectroscopic data was evaluated. Subsequently, for each experiment, PLS models were generated based on spectral data of two cultivation conditions. Internal and external model validation was conducted using data not used for calibration. In a direct comparison with the results from a glycerol variation study, a decreased PLS model performance was observed, which was attributed to an altered carbon flux. However, the calculated RMSEs were comparable to stirred tank PLS regression studies found in literature, which underlines the potential of the high-throughput 2D fluorescence spectroscopic monitoring technology. In a final model refinement, additional scattered light exclusion was shown to result in a change in the dominating spectral dynamics. This resulted in an improved PLS model performance for the pH value of the phosphate limitation experiment. As for the same dataset, the predictability of the glycerol concentration and the CDW did not improve, a high dependence of the modeling results on the input data was suggested.

In conclusion, this study provides a practical example for using the 2D fluorescence monitoring technology in MTPs at elevated throughput. The successful generation and application of PLS models for cultivations with secondary substrate limitations suggest promising applications in early-process development. The obtained information on the apparent metabolism enables the calculation of growth phase-dependent process parameters, such as growth rates, substrate consumption rates, as well as product formation rates. Furthermore, the study supports the previously asserted potential for the reduction of sampling efforts during fermentation. In future research, the acquired datasets can be used for developing more systematic data evaluation workflows for cultivations in the 2D fluorescence online monitoring system.

## Methods

### Microorganisms

The green fluorescent protein (GFP) expressing *Hansenula polymorpha* RB11 pC10-*FMD* (P_*FMD*-GFP_) strain was used for all experiments. For storage at − 80 °C, cryo cultures were prepared in a modified SYN6-MES medium and supplemented with 150 g/L glycerol.

### Media composition

#### Preparation of fully supplemented media

Analogue to a previous study [41], a modified SYN6-MES mineral medium based on Jeude et al. [53] was used for pre and main cultures. The basal solution comprised 1.0 g/L KH_2_PO_4_, 7.66 g/L (NH_4_)_2_SO_4_, 27.3 g/L (140 mM) 2-morpholinoethanesulfonic acid (MES), 3.0 g/L MgSO_4_·7H_2_O, 3.3 g/L KCl and 0.3 g/L NaCl. The pH was adjusted to 6.0 using 1 M NaOH. After sterilisation at 121 °C for 20 minutes, a sterile-filtered trace element solution was supplemented to provide final concentrations of 0.65 mg/L NiSO_4_·6H_2_O, 0.65 mg/L CoCl_2_·6H_2_O, 0.65 mg/L H_2_BO_4_, 0.65 mg/L KI and 0.65 mg/L Na_2_MoO_4_·2H_2_O. A sterile microelement solution was added to provide 66.5 mg/L EDTA (Titriplex III), 66.5 mg/L (NH_4_)_2_Fe(SO_4_)_2_·6H_2_O, 5.5 mg/L CuSO_4_·5H_2_O, 20 mg/L ZnSO_4_·7H_2_O and 26.5 mg/L MnSO_4_·H_2_O. Additionally, a sterile-filtered stock solution was prepared to supplement 1.0 g/L CaCl_2_·2H_2_O. A sterile vitamin solution was added to supply 0.4 mg/L D-biotin and 133.4 mg/L thiamine hydrochloride. For preparing the vitamin stock solution, the D-biotin was first dissolved in a 10 mL mixture (1:1) of 2-propanol and deionised water. The thiamine hydrochloride was dissolved in 90 ml of deionised water and mixed with the D-biotin solution. Next, glycerol was added to the media from a sterile 500 g/L stock solution to achieve the final glycerol concentrations. Finally, sterile water was added to account for differences in volumes.

#### Preparation of secondary substrate limited media solutions

Media with reduced secondary substrate concentrations of magnesium (Mg^2+^), potassium (K^+^) and phosphate (PO_4_^3−^) were prepared according to Kottmeier et al. [47]. For each experiment, the basal medium was mixed with a medium lacking the respective secondary substrate to achieve the desired media composition.

For preparing the medium without magnesium, the supplemented MgSO_4_·7H_2_O was replaced by 1.81 g/L Na_2_SO_4_. The final magnesium concentrations, used during cultivations, accounted for 1.8% (5.32 mg/L), 1.4% (4.14 mg/L), 0.8% (2.37 mg/L), 0.4% (1.18 mg/L) and 0% (0.0 mg/L) of the standard magnesium concentration of the modified SYN6-MES medium (100%, 295.8 mg/L).

For the medium without potassium, KH_2_PO_4_ and KCl were replaced by 0.845 g/L (NH_4_)H_2_PO_4_ to avoid phosphate limitation. 2.92 g/L NaCl was added to avoid changes in osmolality. The final potassium concentrations held for 5% (100.9 mg/L), 4% (80.7 mg/L), 3% (60.5 m/L), 2.5% (50.4 mg/L), 1.75% (35.3 mg/L), 1.25% (25.2 mg/L), 1% (20.2 mg/L) and 0% (0.0 mg/L) of the standard potassium concentration of the SYN6-MES medium (100%, 2017.3 mg/L).

For the medium without phosphate, KH_2_PO_4_ was replaced by 0.547 g/L KCl to avoid a potassium limitation. The final phosphate concentrations held for 35% (244.3 mg/L), 20% (139.6 mg/L), 15% (104.7 mg/L), 12.5% (87.2 mg/L), 10% (69.8 mg/L), 7.5% (52.3 mg/L) and 0% (0.0 mg/L) of the fully supplemented modified SYN6-MES medium (100%, 697.9 mg/L).

### Precultures

Precultures were conducted in 250 mL shake flasks at a filling volume (V_L_) of 10 mL modified SYN6-MES medium supplemented with 10 g/L glycerol. The shaking frequency (n) was chosen to be 350 rpm at a shaking diameter (d_0_) of 50 mm. The temperature (T) was set to 30 °C. The medium was inoculated from the cryo cultures at an initial optical density measured at a wavelength of 600 nm (OD_600_) of 0.1. The precultures were harvested after the OTR indicated no further growth (Additional file 1: Fig. S12). After that, the cells were centrifuged and thoroughly washed with either magnesium-free, potassium-free, or phosphate-free basal medium solution before being added to the main culture media.

### Main cultures

The method for the cultivation of the main cultures was identical to Berg et al. [41]. The cultivations were conducted in three devices (Additional file 1: Fig. S1, dashed boxes) using up to five 48-round well MTPs with a transparent bottom (MTP-R48-B, Beckman Coulter GmbH**,** Aachen, Germany). Identical inoculation conditions were ensured by preparing one inoculated stock solution per initial cultivation condition, as indicated by different colours in Additional file 1: Fig. S1. In all devices, the cultures were continuously shaken at a shaking frequency (n) of 1000 rpm, a shaking diameter (d_0_) of 3 mm and a filling volume (V_L_) of 0.8 mL. The cultivation temperature was set to 30 °C.

### Online monitoring

Multi-wavelength 2D fluorescence online monitoring was conducted in the MTP cultivation platform described by Ladner et al. [12] (Additional file 1: Fig. S1B). For generating 2D spectra, the excitation wavelengths (λ_ex_) were scanned from 280 to 700 nm with an increment of 10 nm. For each excitation wavelength, an emission spectrum, ranging from 275 to 725 nm at a resolution of 0.44 nm, was recorded, using an integration time of 30 ms. After completing the recording of a 2D spectrum for all monitored wells, an intermediate 10 min measurement break was included, to reduce the mechanical wearing of the device. During the measurement breaks, cultivation conditions were maintained. Although not all wells of the 48-well MTPs were exploited in this study, still, 2D-spectra were recorded. As a result, 2D spectra were measured every 30 min for each well of the 48-well MTP.

For determining the OTRs of the individual cultivation conditions, the Respiration Activity MOnitoring System (μRAMOS, Flitsch et al., 2016) was used (Additional file 1: Fig. S1A). The oxygen partial pressure (pO_2_) of the gas phase in the headspace of every individual well was measured by oxygen-sensitive fluorescence spots, using the Stern-Volmer equation for quenching. Subsequently, the OTR was calculated according to Flitsch et al. [48].

### Offline analytics

The MTPs (MTP-R48-OFF, Beckman Coulter, Aachen, Germany) used for sampling (Additional file 1: Fig. S1C) were incubated in a separate humidified incubator (ISF1-X, Adolf Kühner AG, Birsfelden, Switzerland). The exact sampling time points accounted for a sampling interval as low as 1 h. In total, 144 (Mg^2+^), 128 (K^+^) and 122 (PO_4_^3−^) samples were taken from the three experiments, respectively. At each sampling time, the culture broth was manually withdrawn from one well per sampled condition using a micro pipette. Due to the small shaking diameter, the orbital shaking was maintained during the sampling. Thereby, oxygen limitation was avoided [35, 68]. The initial samples (t = 0 h) were taken from the prepared inoculated medium of the main cultures. The OD_600_ of the samples was determined using a photometer (GENESYS 20, Thermo Scientific, Dreieich, Germany) and micro cuvettes (PS, Carl Roth, Karlsruhe, Germany). Measurements between 0.1 and 0.3 a.u. were ensured by sample dilution with a 0.9% (w/v) NaCl solution. The cell dry weight (CDW) was calculated using a linear correlation with the OD_600_ (Additional file 1: Fig. S13). The pH value was determined using a two-point calibrated pH meter (HI21, HANNA instruments Inc., Woonsocket, US, electrode: InLab Solids, Mettler Toledo GmbH, Columbus, US). For pH measurements, undiluted culture broth samples were used. The glycerol concentration was determined by HPLC measurements, following the protocol of Berg et al. [41].

### Spectral data processing and multivariate data analysis

The spectral data processing and MVDA were identical to the method described by Berg et al. [41], using MATLAB 9.11.0.1769968 (R2021b) and the open-source toolbox *mdatools* [69]. The raw 2D spectra were initially reduced to include only emission wavelengths (λ_em_) between − 10 nm and + 270 nm relative to the excitation wavelength. A moving average filter with a window size of 25 pixels (equivalent to 11.45 nm) was applied for noise reduction. The spectral data was further reduced to a resolution of 2 nm to reduce the processing time. No significant deteriorating effect on the modeling results was observed by this procedure (data not shown). For excluding the scattered light in silico, the intensities between − 10 nm and + 13 nm relative to the excitation wavelength were discarded. Finally, for each well, the spectral data of each measurement cycle was referenced to the respective first 2D spectrum by subtraction per wavelength combination (I-I_0_).

The temporal alignment of the online spectral data (Additional file 1: Fig. S1B) and the offline measurements (Additional file 1: Fig. S1C) was conducted by linear interpolation of the latter. Subsequently, for each time of spectral measurement, the interpolated offline parameter values were extracted from the interpolation. For PLS model generation, the calibration dataset consisted of spectral replicates of two cultivation conditions, including one high and one low secondary substrate supplementation. The linear interpolation of the calibration datasets included between six and eight samples with a sampling interval of at least 5 h. For validating the models, the linear interpolation included all available samples leading to a sampling interval as low as 1 h. The prediction dataset consisted of spectral data of six cultivation conditions not used for calibration. Here, the linear interpolation included all available offline samples. The PLS models were generated for each offline parameter using the SIMPLS algorithm [70] before being evaluated by calculating the root-mean-square error (RMSE).

## Supplementary Information

**Additional file 1: Fig. S1.** Overview of experimental monitoring and sampling strategy used in the cultivation experiments of this study. Three parallel cultivation devices (dashed boxes) were used for online (Fig. S1A and B, cross-hatched wells) and offline (Fig. S1C, unhatched wells) monitoring of the cultures of eight different initial cultivation conditions (I–VIII). Grey hatched wells show wells filled with non-inoculated, fully supplemented medium for control. Struck-through wells were not used in the experiment. (**A**) For the limitation studies of magnesium and phosphate, a μRAMOS cultivation system as described by Flitsch et al. [48] was used, to monitor the metabolic activity in form of the oxygen transfer rate (OTR). The number of replicates varied depending on the experiment (Fig. S3 and S8). (**B**) Online 2D fluorescence data was generated using the online monitoring cultivation system published by Ladner et al. [12]. (**C**) Offline samples were taken in singlets from three additional microtiter plates (MTPs). The total number of offline samples varied depending on the experiment and cultivation condition. Adapted from Berg et al. [41]. **Fig. S2.** Exemplary 2D spectra recorded at different cultivation times for *H. polymorpha* RB11 pC9-*FMD* (P_*FMD*_-GFP) cultivated at two different initial cultivation conditions and non-inoculated medium. Spectra recorded for cultures cultivated with a CDW_t0_ of 0.03 g/L and initial magnesium (Mg^**2**+^) concentrations of 295.8 mg/L and 2.37 mg/L are shown in A-D (light blue rectangle) and E-H (orange rectangle), respectively. I-L (black rectangle) shows spectra for non-inoculated medium. Spectra are shown for cultivation times of (**A, E, I**) 0 h, **(B, F, J**) 12 h, (**C, G, K**) 24 h, and (**D, H, L**) 30 h. Spectroscopic measurement settings: excitation wavelength range = 280 nm – 700 nm (step size = 10 nm), emission wavelength range = 278 nm – 720 nm (step size = 0.45 nm), integration time = 30 ms. Cultivation conditions: 48-well microtiter plate with round well geometry, modified SYN6-MES medium, liquid volume = 800 μL, shaking diameter = 3 mm, shaking frequency = 1000 rpm, temperature = 30 °C. **Fig. S3.** Time-resolved oxygen transfer rate (OTR) signals of individual *H. polymorpha* RB11 pC9-*FMD* (P_*FMD*_-GFP) cultivations at different initial cell dry weight (CDW_t0_) and magnesium (Mg^**2**+^) concentrations. Solid, dashed, and dotted lines describe the OTR of individual cultures used for calculating the average and standard deviation shown in Fig. 1A. The number of replicates (n) is shown in the legend. Cultivation conditions: 48-well microtiter plate with round geometry, modified SYN6-MES medium, liquid volume = 800 μL, shaking diameter = 3 mm, shaking frequency = 1000 rpm, temperature = 30 °C. **Fig. S4.** Impact of the number of latent variables on the root-mean-square error (RMSE) for the 2D spectra-based PLS models of (**A**) glycerol, (**B**) cell dry weight (CDW) and (**C**) pH of the magnesium variation experiment. The PLS models are based on the data in Fig. 1. All PLS models were calibrated using offline values from the linearly interpolated, 6 h sampling interval (Fig. 1D-F, filled symbols) from which the RMSE_Cal, sparse_ (black circles) was calculated. The RMSE_Cal, full_ (red upward triangles) was calculated from the 1.5 h sampling interval (Fig. 1D-F, filled and hollow symbols). The RMSE_Pred, full_ (green downward triangles) was calculated for the prediction dataset, and the 1.5 h sampling interval was based on the offline values (Fig. 2D-F, hollow symbols). **Fig. S5.** Time-resolved (**A-B**) online monitoring signals and (**C-E**) offline sample measurements of *H. polymorpha* RB11 pC9-*FMD* (P_*FMD*_-GFP) cultivations at different potassium (K^+^) concentrations. (**A**) Scattered light intensities (λ_ex _= λ_em_ = 600 nm) and (**C**) GFP fluorescence intensities (λ_ex_ = 420 nm, λ_em_ = 530 nm) were extracted from 2D spectra of duplicates, shown as solid and dotted lines. Hollow symbols indicate every 10th data point. Values of (**C**) glycerol, (**D**) cell dry weight (CDW), and (**E**) pH value for cultures with an initial potassium concentration of 100.9 mg/L (blue upward triangles) and 20.2 mg/L (pink squares), respectively, are based on singular offline measurements. Hollow symbols show offline measurements for the short sampling interval. Filled, linearly interpolated symbols describe a sparse, more realistic sampling interval of at least 5 h. The vertical dashed line after 43 h describes the last measurement included for PLS modeling. Cultivation conditions: 48-well microtiter plate with round geometry, modified SYN6-MES medium, liquid volume = 800 μL, shaking diameter = 3 mm, shaking frequency = 1000 rpm, temperature = 30 °C. **Fig. S6.** Impact of the number of latent variables on the root-mean-square error (RMSE) for the 2D spectra-based PLS models of (**A**) glycerol, (**B**) cell dry weight (CDW) and (**C**) pH of the potassium variation experiment. The PLS models are based on the data in Fig. 1. All PLS models were calibrated using offline values from the linearly interpolated, sparse sampling interval (Fig. S5C-E, filled symbols), from which the RMSE_Cal, sparse_ (black circles) was calculated. The RMSE_Cal, full_ (red upward triangles) was calculated based on the linear interpolation of all available offline samples (Fig. S5C-E, filled and hollow symbols). The RMSE_Pred, full_ (green downward triangles) was calculated for the prediction dataset and the respective offline values (Fig. 3D-F, hollow symbols). For the RMSE_Pred, full, − 100%_ (blue diamonds), the cultures with 2017.3 mg/L potassium (Fig. S5, purple circles) were excluded from the calculation. **Fig. S7.** Time-resolved (**A-C**) online monitoring signals and (**D-F**) offline sample measurements of *H. polymorpha* RB11 pC9-*FMD* (P_*FMD*_-GFP) cultivations at different phosphate (PO_4_^3−^) concentrations. (**A**) The mean oxygen transfer rate (OTR) of culture replicates (*n* = 2–3, Additional file 1: Fig. S8) was determined by a μRAMOS device [48]. The low standard deviations are shown as shaded areas and indicate good reproducibility. Hollow symbols indicate every 15th data point. (**B**) Scattered light intensities (λ_ex_ = λ_em_ = 600 nm) and (**C**) GFP fluorescence intensities (λ_ex_ = 420 nm, λ_em_ = 530 nm) were extracted from 2D spectra of duplicates, shown as solid and dotted lines. Hollow symbols indicate every fifth data point. Values of (**D**) glycerol, (**E**) cell dry weight (CDW), and (**F**) pH value for cultures with an initial phosphate concentration of 244.3 mg/L (blue upward triangles) and 52.3 mg/L (red downward triangles), respectively, are based on singular offline measurements. Hollow symbols show offline measurements for the short sampling. Filled, linearly interpolated symbols describe a sparse, sampling interval of at least 5 h. The vertical dashed line after 34.5 h describes the last measurement included for PLS modeling. Cultivation conditions: 48-well microtiter plate with round geometry, modified SYN6-MES medium, liquid volume = 800 μL, shaking diameter = 3 mm, shaking frequency = 1000 rpm, temperature = 30 °C. **Fig. S8.** Time-resolved oxygen transfer rate (OTR) signals of individual *H. polymorpha* RB11 pC9-*FMD* (P_*FMD*_-GFP) cultivations at different phosphate (PO_4_^3−^) concentrations. Solid, dashed, and dotted lines describe the OTR of individual cultures used for calculating the average and standard deviation, shown in Fig. S7A. The number of replicates (n) is shown in the legend. Cultivation conditions: 48-well microtiter plate with round geometry, modified SYN6-MES medium, liquid volume = 800 μL, shaking diameter = 3 mm, shaking frequency = 1000 rpm, temperature = 30 °C. **Fig. S9.** Impact of the number of latent variables on the root-mean-square error (RMSE) for the 2D spectra-based PLS models of (**A**) glycerol, (**B**) cell dry weight (CDW) and (**C**) pH of the phosphate variation experiment. Resulting errors are shown for PLS models using the spectral dataset (**A-C**) including the scattered light and fluorescence, and (**D-F**) including only the fluorescence. All PLS models were calibrated using offline values from the linearly interpolated, sparse sampling interval (Fig. S7D-F, filled symbols), from which the RMSE_Cal, sparse_ (black circles) was calculated. The RMSE_Cal, full_ (red upward triangles) was calculated based on the linear interpolation of all available offline samples (Fig. S7D-F, filled and hollow symbols). The RMSE_Pred, full_ (green downward triangles) was calculated for the prediction dataset and the respective offline values (Fig. 4E-H, hollow symbols). For the calculation of the RMSE_Pred, full, − 100%_ (blue diamonds), the cultures with 697.9 mg/L phosphate (Fig. 4E-H, Fig. S7D-F, purple circles) were excluded. **Fig. S10.** Exemplary 2D spectra of *H. polymorpha* RB11 pC9-*FMD* (P_*FMD*_-GFP) cultures after 24 h of cultivation cultivated at initial phosphate (PO_4_^3−^) concentrations of (**A, C**) 697.9 mg/L and (**B, D**) 52.3 mg/L. Spectra (**A, B**) including scattered light and fluorescence, as well as (**C, D**) spectra including fluorescence only are shown. The scattered light exclusion was conducted in silico*,* as described in the material and methods section. Spectroscopic measurement settings: excitation wavelength range = 280 nm – 700 nm (step size = 10 nm), emission wavelength range = 278 nm – 720 nm (step size = 0.45 nm), integration time = 30 ms. Cultivation conditions: 48-well microtiter plate with round geometry, modified SYN6-MES medium, liquid volume = 800 μL, shaking diameter = 3 mm, shaking frequency = 1000 rpm, temperature = 30 °C. **Fig. S11.** Comparison of absolute RMSE_Pred, full_ for glycerol, CDW and pH value for the PLS models generated in Berg et al. [41] and this study. Plain columns show the RMSE based on the complete prediction datasets from this study and the study by Berg et al. [41]. Backward diagonal hatched columns describe the RMSE based on the complete prediction dataset, except the culture holding the initial concentration of the respective second substrate (− 100%). Forward-hatched columns describe the RMSE calculated for spectral online datasets including fluorescence (Fl) only. For the diagonal cross-hatched columns, additionally, the cultures with the initial concentration of the respective second substrate, according to Jeude et al. [53], were excluded (− 100%). Exact values as well as the number of LVs used for each model are summarized in Additional file 2**:** Table S1. **Fig. S12.** Exemplary Oxygen transfer rate (OTR) measured for the preculture of *Hansenula polymorpha* RB11 pC9-*FMD* (P_*FMD*_-GFP). Cultivation conditions: modified SYN6-MES medium, 10 g/L glycerol, initial optical density = 0.1, 250 mL shake flask, filling volume = 10 mL, shaking frequency = 350 rpm, shaking diameter = 50 mm, T = 30 °C. **Fig. S13.** Linear correlation between cell dry weight (CDW) and optical density at 600 nm (OD_600_). Cultures were grown in a modified SYN6-MES medium according to the preculture protocol. The shake flask cultures were harvested during exponential growth before the dilution series were prepared with fresh medium. For the OD_600_ measurement of the diluted series, additional dilution was conducted to allow measurements between 0.1 and 0.3. Adapted from Berg et al. [41].

**Additional file 2: Table S1.** Absolute and relative RMSE of calibration and prediction for PLS models of glycerol, CDW, and pH-value. Results are shown for the experiments of this study and the previous study for glycerol variation [41]. The RMSEs for calibration and prediction are shown for the shortest available sampling interval (RMSE_Cal,full_, RMSE_Pred,full_). The relative RMSE, shown in brackets, is calculated based on the offline parameter range for the respective experiment.

## Data Availability

The datasets used and analysed during the current study are available from the corresponding author upon reasonable request.

## Abbreviations

CCD: charge-coupled device
CDW: cell dry weight
FAD: flavin adenine dinucleotide
FMD: formate dehydrogenase
GFP: green fluorescent protein
λ_em_: emission wavelength
λ_ex_: excitation wavelength
LV: latent variable
MTP: microtiter plate
MVDA: multivariate data analysis
MES: morpholinoethanesulfonic acid
NADH: nicotinamide adenine dinucleotide
OTR: oxygen transfer rate
OD: optical density
PC: principal component
PCA: principal component analysis
PLS: partial least square;
pO_2_: oxygen partial pressure
RAMOS: Respiration Activity MOnitoring System
RMSE: root-mean-square error
