# Supplementary material for: Assessing the capabilities of 2D fluorescence monitoring in microtiter plates with data-driven modeling for secondary substrate limitation experiments of Hansenula polymorpha
Source: J Biol Eng. 2023 Feb 13;17:12. doi: 10.1186/s13036-023-00332-0 (PMC9926666; doi:10.1186/s13036-023-00332-0)
Supplement: Supplementary file 1 — Additional file 1: Fig. S1. Overview of experimental monitoring and sampling strategy used in the cultivation experiments of this study. Three parallel cultivation devices (dashed boxes) were used for online (Fig. S1A and B, cross-hatched wells) and offline (Fig. S1C, unhatched wells) monitoring of the cultures of eight different initial cultivation conditions (I–VIII). Grey hatched wells show wells filled with non-inoculated, fully supplemented medium for control. Struck-through wells were not used in the experiment. (A) For the limitation studies of magnesium and phosphate, a μRAMOS cultivation system as described by Flitsch et al. [48] was used, to monitor the metabolic activity in form of the oxygen transfer rate (OTR). The number of replicates varied depending on the experiment (Fig. S3 and S8). (B) Online 2D fluorescence data was generated using the online monitoring cultivation system published by Ladner et al. [12]. (C) Offline samples were taken in singlets from three additional microtiter plates (MTPs). The total number of offline samples varied depending on the experiment and cultivation condition. Adapted from Berg et al. [41]. Fig. S2. Exemplary 2D spectra recorded at different cultivation times for H. polymorpha RB11 pC9-FMD (PFMD-GFP) cultivated at two different initial cultivation conditions and non-inoculated medium. Spectra recorded for cultures cultivated with a CDWt0 of 0.03 g/L and initial magnesium (Mg2+) concentrations of 295.8 mg/L and 2.37 mg/L are shown in A-D (light blue rectangle) and E-H (orange rectangle), respectively. I-L (black rectangle) shows spectra for non-inoculated medium. Spectra are shown for cultivation times of (A, E, I) 0 h, (B, F, J) 12 h, (C, G, K) 24 h, and (D, H, L) 30 h. Spectroscopic measurement settings: excitation wavelength range = 280 nm – 700 nm (step size = 10 nm), emission wavelength range = 278 nm – 720 nm (step size = 0.45 nm), integration time = 30 ms. Cultivation conditions: 48-well microtiter plate with r [file 13036_2023_332_MOESM1_ESM.pdf]

Supplementary for research article

Assessing the capabilities of 2D fluorescence monitoring in microtiter plates with data-driven modeling for secondary substrate limitation experiments of *Hansenula polymorpha*

Christoph Berg [a], Laura Herbst [a], Lisa Gremm [a], Nina Ihling [a], Olivier Paquet-Durand [b], Bernd Hitzmann [b], Jochen Büchs [a]

[a] AVT - Aachener Verfahrenstechnik, Biochemical Engineering, RWTH Aachen University, Forckenbeckstraße 51, 52074 Aachen, Germany;

[b] Department of Process Analytics & Cereal Science, Institute for Food Science and Biotechnology, University of Hohenheim, Garbenstraße 23, 70599 Stuttgart, Germany

Correspondence: Prof. Dr.-Ing. Jochen Büchs (Jochen.Buechs@avt.rwth-aachen.de)

AVT - Biochemical Engineering, RWTH Aachen University, Forckenbeckstraße 51, 52074 Aachen, Germany.

Keywords: 2D Fluorescence spectroscopy, Online monitoring, Multivariate data analysis, High-throughput, Microbioreactor, Microtiter plate, Secondary substrate limitation, *Hansenula polymorpha*

Abbreviations: CCD: charge-coupled device; **CDW**: cell dry weight; **FAD**: flavin adenine dinucleotide; **FMD**: formate dehydrogenase; **GFP**: green fluorescent protein; $\boldsymbol{\lambda_{em}}$: emission wavelength; $\boldsymbol{\lambda_{ex}}$: excitation wavelength; **LV**: latent variable; **MTP**: microtiter plate; **MVDA**: multivariate data analysis; **MES**: morpholinoethanesulfonic acid; **NADH**: nicotinamide adenine dinucleotide; **OTR**: oxygen transfer rate; **OD**: optical density; **PC**: principal component; **PCA**: principal component analysis; **PLS**: partial least square; **pO2**: oxygen partial pressure; **R²**: correlation coefficient; **RAMOS**: Respiration Activity MOnitoring System; RMSE: root-mean-square error

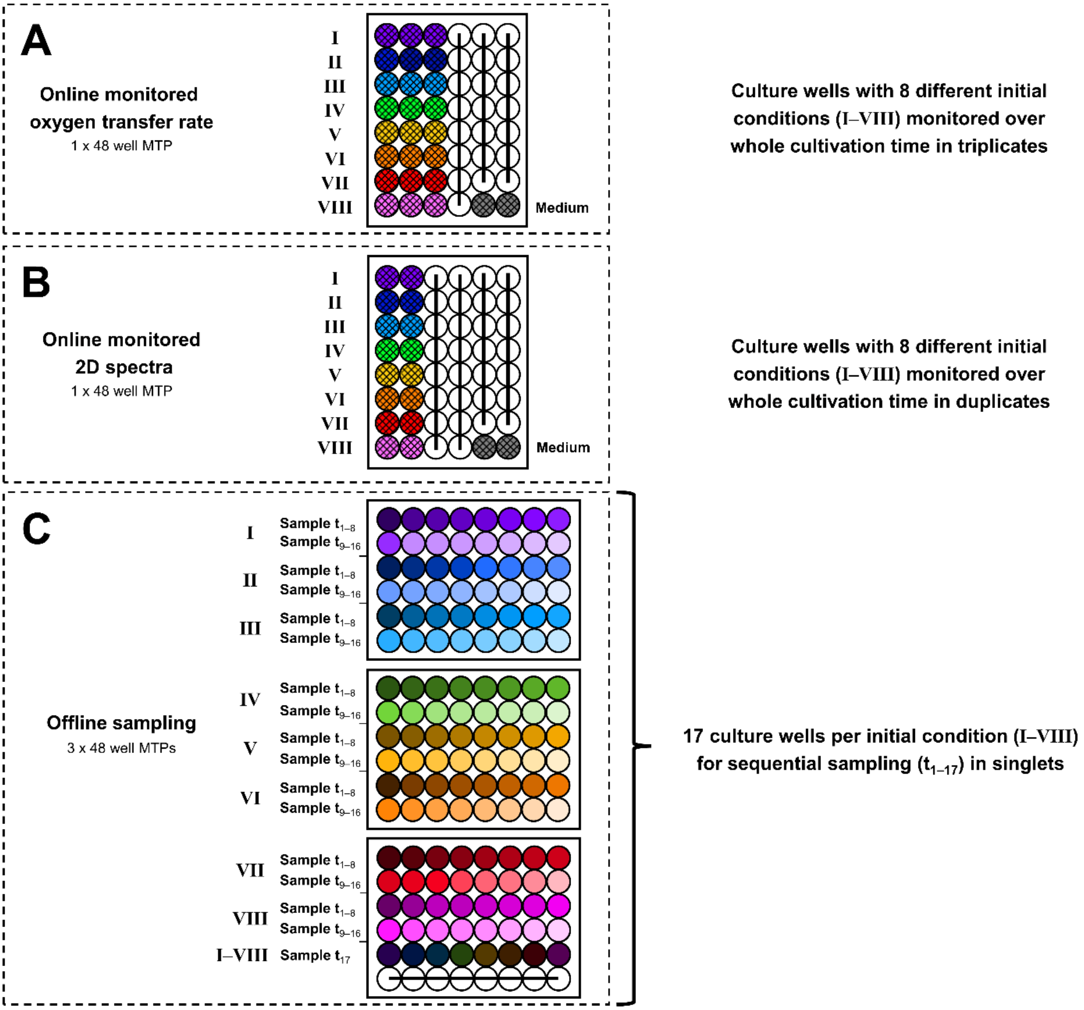

Figure S1: Overview of experimental monitoring and sampling strategy used in the cultivation experiments with secondary substrate limitation of this study. Three parallel cultivation devices (dashed boxes) were used for online (Figure S1A and B, cross-hatched wells) and offline (Figure S1C, unhatched wells) monitoring of the cultures of eight different initial cultivation conditions (I–VIII). Grey hatched wells show wells filled with non-inoculated, fully supplemented medium for control. Struck-through wells were not used in the experiment. (A) For the limitation studies of magnesium and phosphate, a µRAMOS cultivation system as described by Flitsch et al. [67] was used, to monitor the metabolic activity in form of the oxygen transfer rate (OTR). The number of replicates varied depending on the experiment (Figure S3 and S8). (B) Online 2D fluorescence data was generated using the online monitoring cultivation system published by Ladner et al. [12]. (C) Offline samples were taken in singlets from three additional microtiter plates (MTPs). The total number of offline samples varied depending on the experiment and cultivation condition. Adapted from Berg et al. [41].

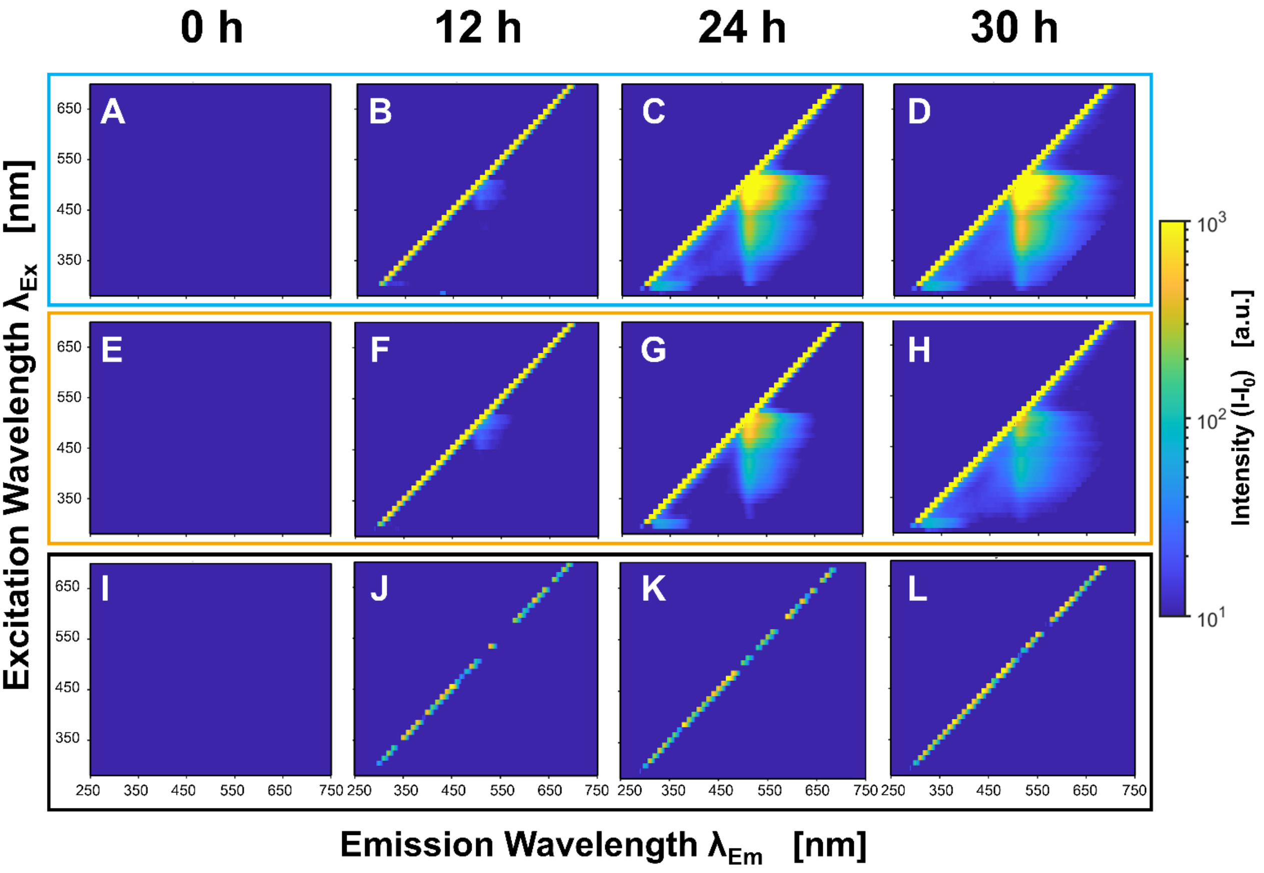

Figure S2: Exemplary 2D spectra recorded at different cultivation times for *H. polymorpha* RB11 pC9-*FMD* (P_{FMD}-GFP) cultivated at two different initial cultivation conditions and non-inoculated medium. Spectra recorded for cultures cultivated with a CDW_{t0} of 0.03 g/L and initial magnesium (Mg^{2+}) concentrations of 295.8 mg/L and 2.37 mg/L are shown in A-D (light blue rectangle) and E-H (orange rectangle), respectively. I-L (black rectangle) shows spectra for non-inoculated medium. Spectra are shown for cultivation times of (A, E, I) 0 h, (B, F, J) 12 h, (C, G, K) 24 h, and (D, H, L) 30 h. Spectroscopic measurement settings: excitation wavelength range = 280 nm – 700 nm (step size = 10 nm), emission wavelength range = 278 nm – 720 nm (step size = 0.45 nm), integration time = 30 ms. Cultivation conditions: 48-well microtiter plate with round well geometry, modified SYN6-MES medium, liquid volume = 800 µL, shaking diameter = 3 mm, shaking frequency = 1000 rpm, temperature = 30 °C

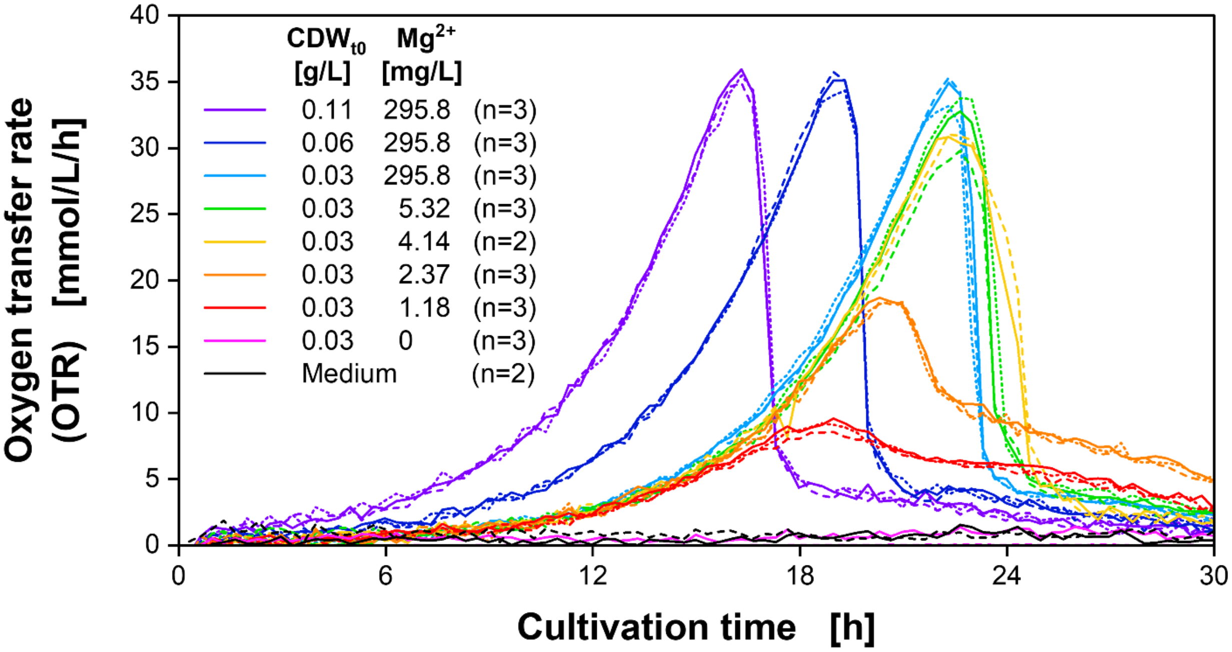

Figure S3: Time-resolved oxygen transfer rate (OTR) signals of individual *H. polymorpha* RB11 pC9-*FMD* (P_{FMD}-GFP) cultivations at different initial cell dry weight (CDW_{t0}) and magnesium (Mg^{2+}) concentrations. Solid, dashed, and dotted lines describe the OTR of individual cultures used for calculating the average and standard deviation shown in Figure 1A. The number of replicates (n) is shown in the legend. Cultivation conditions: 48-well microtiter plate with round geometry, modified SYN6-MES medium, liquid volume = 800 µL, shaking diameter = 3 mm, shaking frequency = 1000 rpm, temperature = 30 °C.

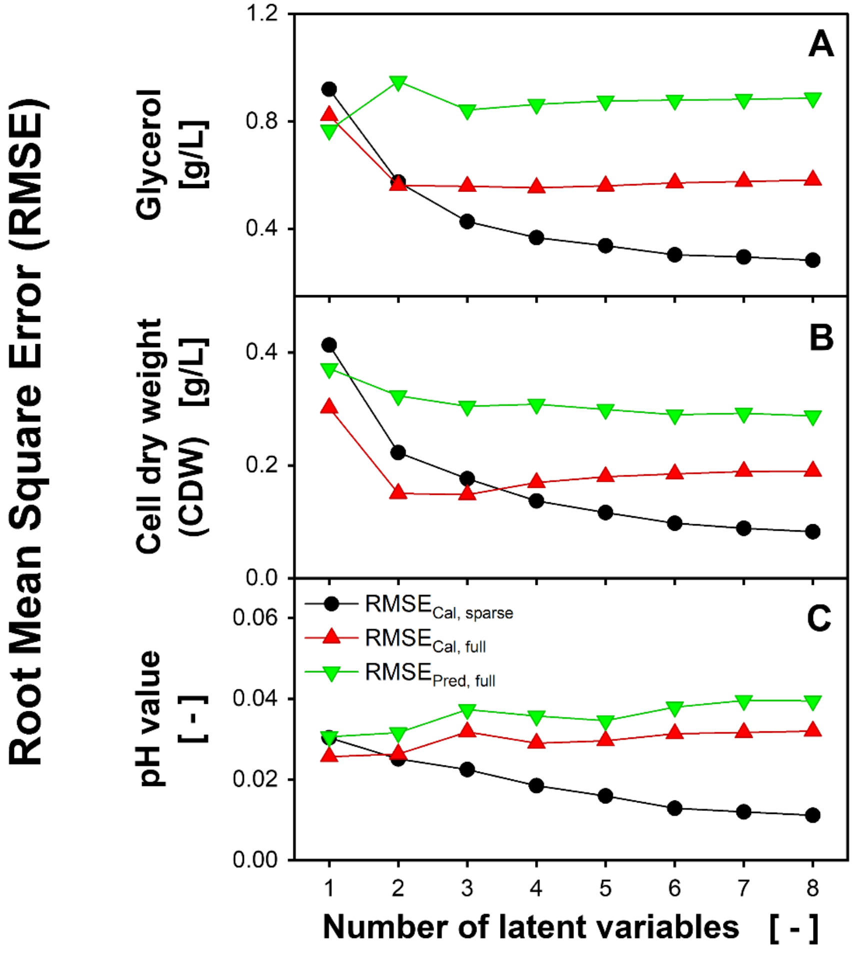

Figure S4: Impact of the number of latent variables on the root-mean-square error (RMSE) for the 2D spectra-based PLS models of (A) glycerol, (B) cell dry weight (CDW) and (C) pH of the magnesium variation experiment. The PLS models are based on the data in Figure 1. All PLS models were calibrated using offline values from the linearly interpolated, 6 h sampling interval (Figure 1D-F, filled symbols) from which the RMSE$_{Cal, sparse}$ (black circles) was calculated. The RMSE$_{Cal, full}$ (red upward triangles) was calculated from the 1.5 h sampling interval (Figure 1D-F, filled and hollow symbols). The RMSE$_{Pred, full}$ (green downward triangles) was calculated for the prediction dataset, and the 1.5 h sampling interval was based on the offline values (Figure 2D-F, hollow symbols).

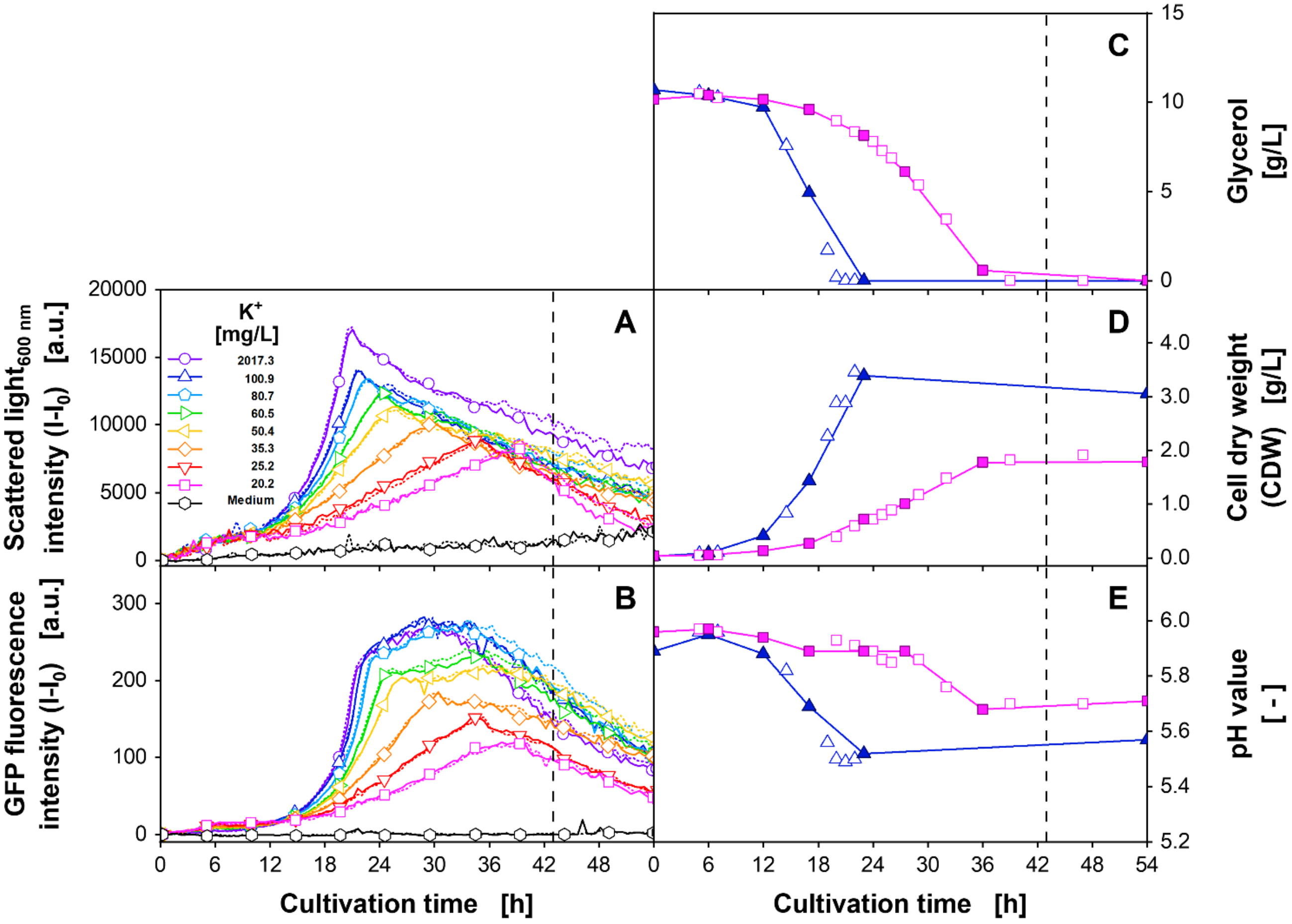

Figure S5: Time-resolved (A-B) online monitoring signals and (C-E) offline sample measurements of *H. polymorpha* RB11 pC9-*FMD* (P_{FMD}-GFP) cultivations at different potassium (K^+) concentrations. (A) Scattered light intensities (λex= λem = 600 nm) and (C) GFP fluorescence intensities (λex= 420 nm, λem= 530 nm) were extracted from 2D spectra of duplicates, shown as solid and dotted lines. Hollow symbols indicate every 10th data point. Values of (C) glycerol, (D) cell dry weight (CDW), and (E) pH value for cultures with an initial potassium concentration of 100.9 mg/L (blue upward triangles) and 20.2 mg/L (pink squares), respectively, are based on singular offline measurements. Hollow symbols show offline measurements for the short sampling interval. Filled, linearly interpolated symbols describe a sparse, more realistic sampling interval of at least 5 h. The vertical dashed line after 43 h describes the last measurement included for PLS modeling. Cultivation conditions: 48-well microtiter plate with round geometry, modified SYN6-MES medium, liquid volume = 800 µL, shaking diameter = 3 mm, shaking frequency = 1000 rpm, temperature = 30 °C.

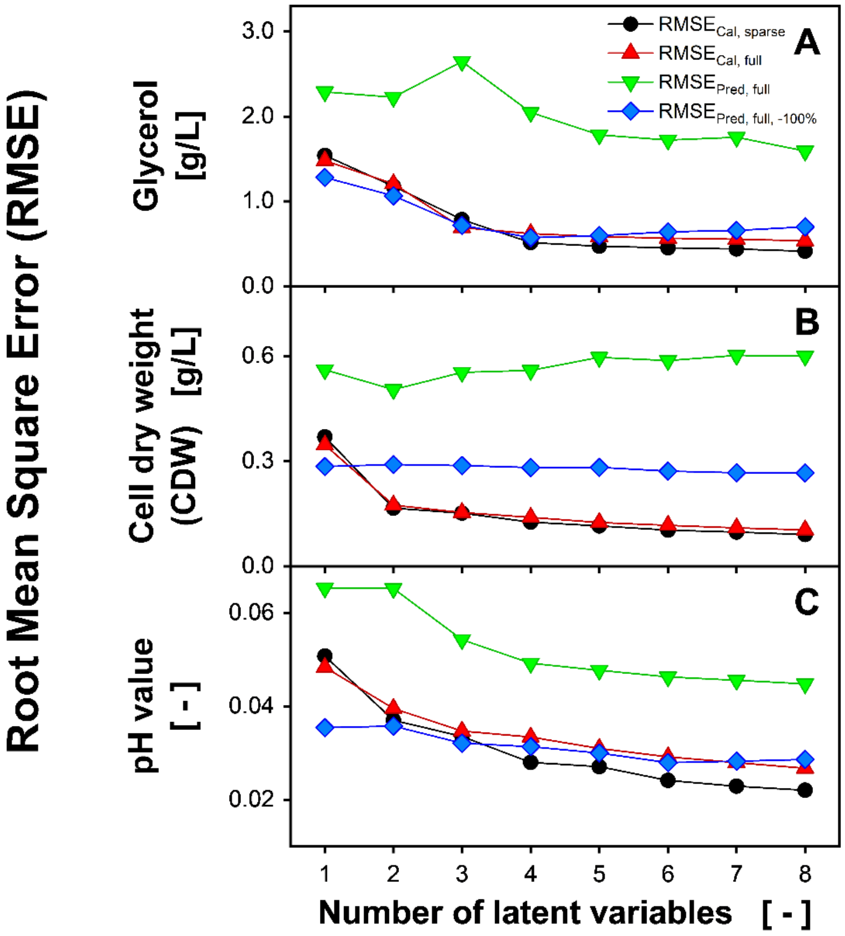

Figure S6: Impact of the number of latent variables on the root-mean-square error (RMSE) for the 2D spectra-based PLS models of (A) glycerol, (B) cell dry weight (CDW) and (C) pH of the potassium variation experiment. The PLS models are based on the data in Figure 1. All PLS models were calibrated using offline values from the linearly interpolated, sparse sampling interval (Figure S5C-E, filled symbols), from which the $RMSE_{Cal, sparse}$ (black circles) was calculated. The $RMSE_{Cal, full}$ (red upward triangles) was calculated based on the linear interpolation of all available offline samples (Figure S5C-E, filled and hollow symbols). The $RMSE_{Pred, full}$ (green downward triangles) was calculated for the prediction dataset and the respective offline values (Figure 3D-F, hollow symbols). For the $RMSE_{Pred, full, -100\%}$ (blue diamonds), the cultures with 2017.3 mg/L potassium (Figure S5, purple circles) were excluded from the calculation.

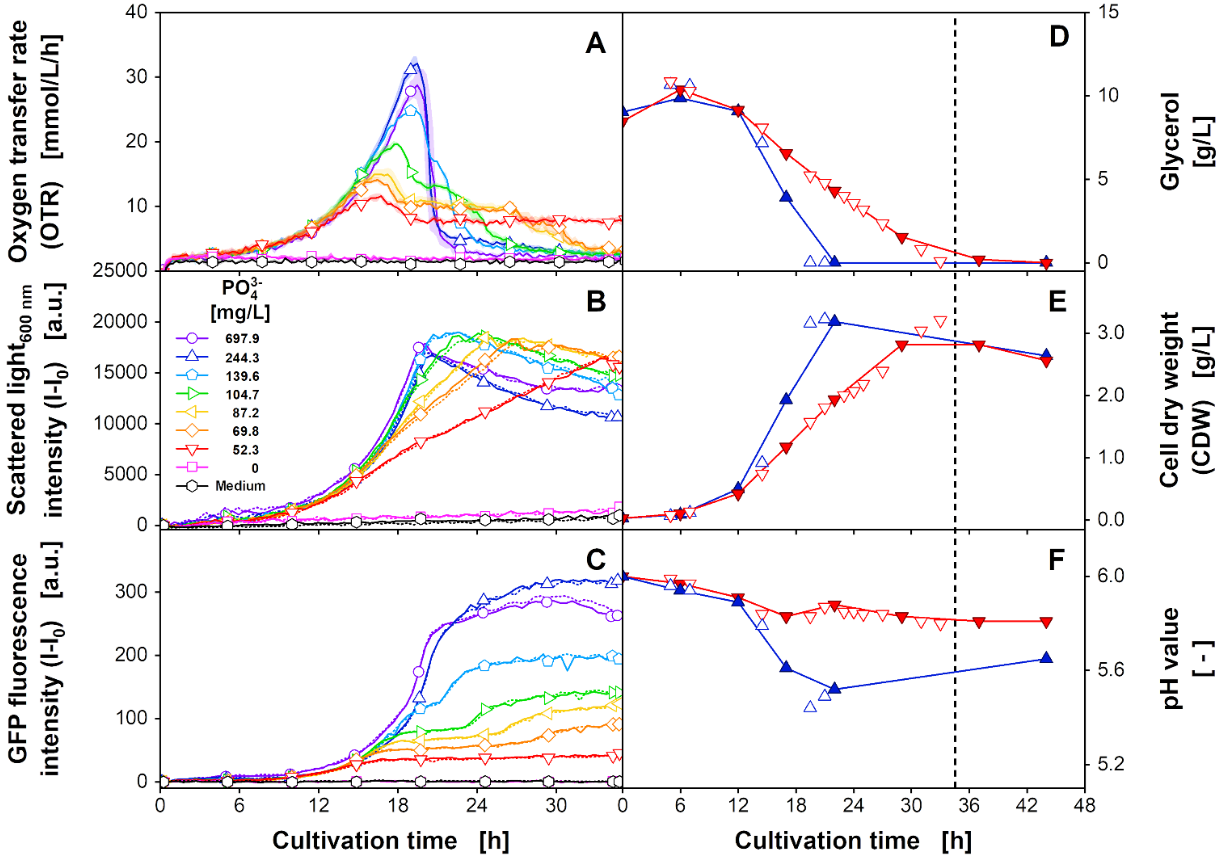

Figure S7: Time-resolved (A-C) online monitoring signals and (D-F) offline sample measurements of *H. polymorpha* RB11 pC9-*FMD* (P_{FMD}-GFP) cultivations at different phosphate (PO) concentrations. (A) The mean oxygen transfer rate (OTR) of culture replicates (n= 2-3, Additional File 1, Figure S8) was determined by a µRAMOS device [67]. The low standard deviations are shown as shaded areas and indicate good reproducibility. Hollow symbols indicate every 15th data point. (B) Scattered light intensities (λ_{ex}= λ_{em} = 600 nm) and (C) GFP fluorescence intensities (λ_{ex}= 420 nm, λ_{em}= 530 nm) were extracted from 2D spectra of duplicates, shown as solid and dotted lines. Hollow symbols indicate every fifth data point. Values of (D) glycerol, (E) cell dry weight (CDW), and (F) pH value for cultures with an initial phosphate concentration of 244.3 mg/L (blue upward triangles) and 52.3 mg/L (red downward triangles), respectively, are based on singular offline measurements. Hollow symbols show offline measurements for the short sampling. Filled, linearly interpolated symbols describe a sparse, sampling interval of at least 5 h. The vertical dashed line after 34.5 h describes the last measurement included for PLS modelling. Cultivation conditions: 48-well microtiter plate with round geometry, modified SYN6-MES medium, liquid volume = 800 µL, shaking diameter = 3 mm, shaking frequency = 1000 rpm, temperature = 30 °C.

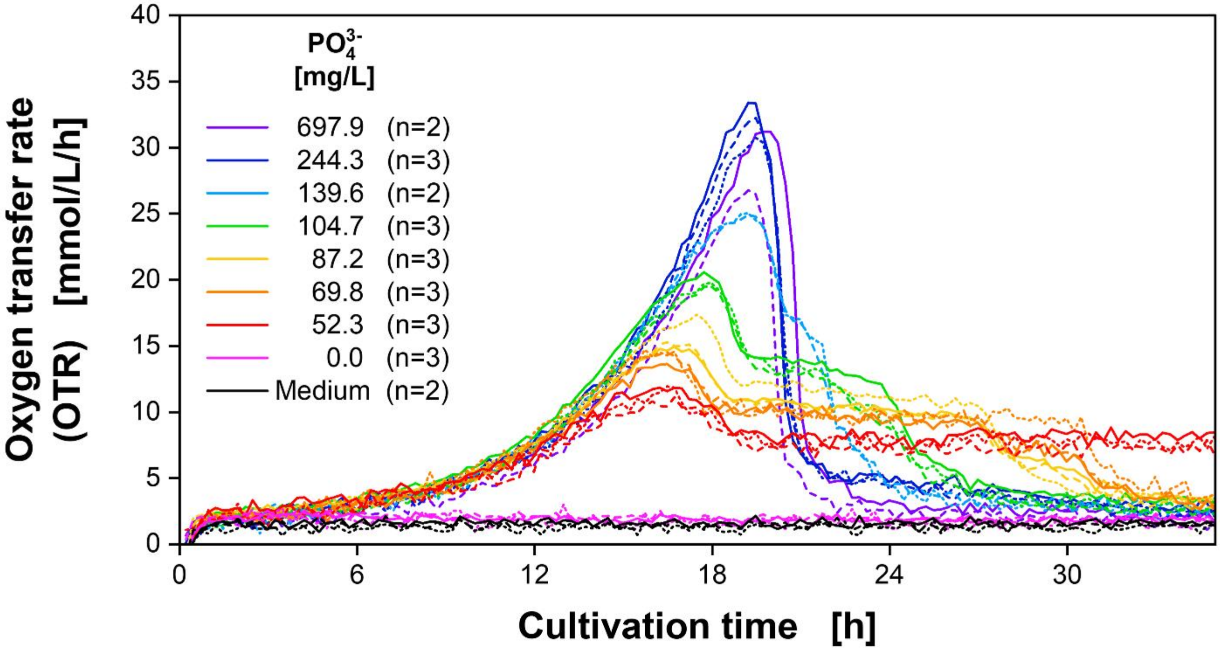

Figure S8: Time-resolved oxygen transfer rate (OTR) signals of individual *H. polymorpha* RB11 pC9-*FMD* (P_{FMD}-GFP) cultivations at different phosphate (PO_4^{3-}) concentrations. Solid, dashed, and dotted lines describe the OTR of individual cultures used for calculating the average and standard deviation, shown in Figure S7A. The number of replicates (n) is shown in the legend. Cultivation conditions: 48-well microtiter plate with round geometry, modified SYN6-MES medium, liquid volume = 800 µL, shaking diameter = 3 mm, shaking frequency = 1000 rpm, temperature = 30 °C.

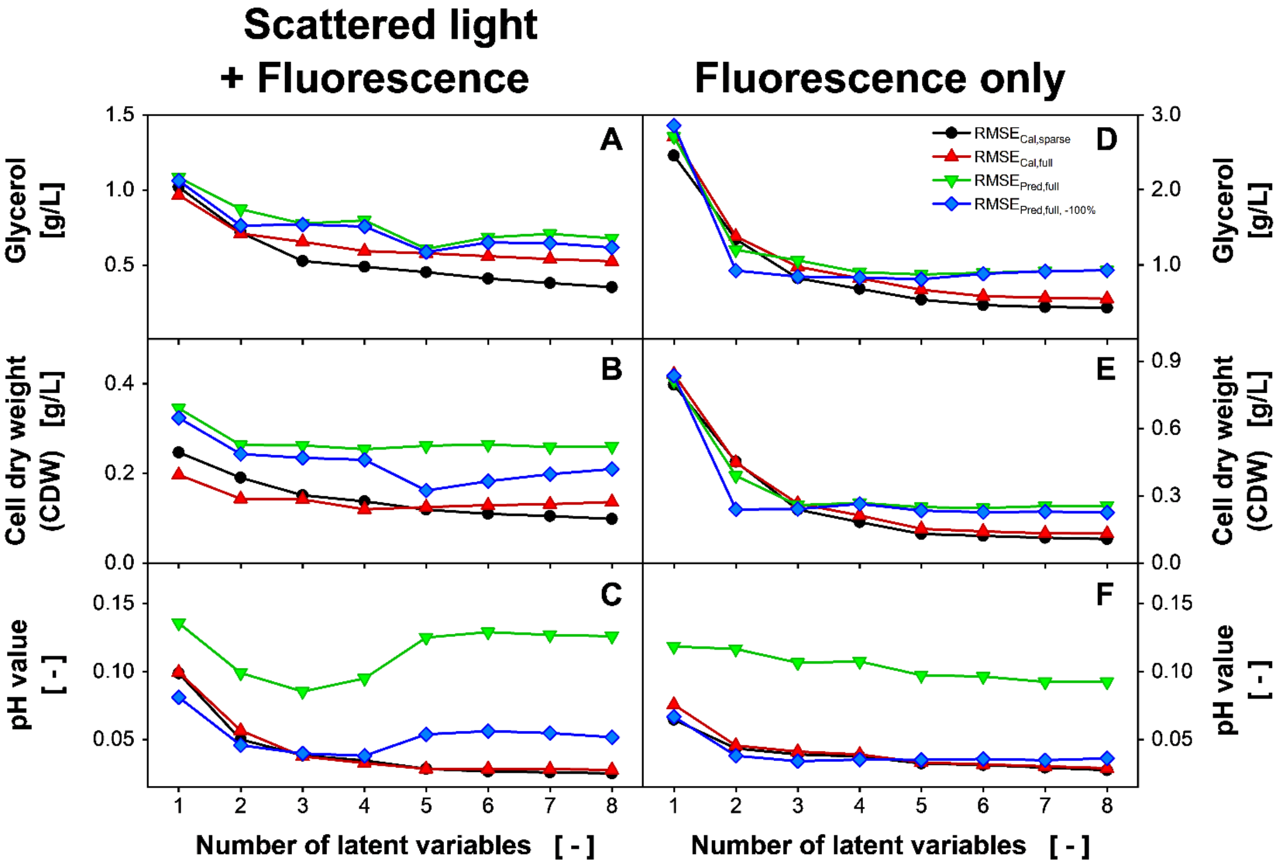

Figure S9: Impact of the number of latent variables on the root-mean-square error (RMSE) for the 2D spectra-based PLS models of (A) glycerol, (B) cell dry weight (CDW) and (C) pH of the phosphate variation experiment. Resulting errors are shown for PLS models using the spectral dataset (A-C) including the scattered light and fluorescence, and (D-F) including only the fluorescence. All PLS models were calibrated using offline values from the linearly interpolated, sparse sampling interval (Figure S7D-F, filled symbols), from which the $RMSE_{Cal, sparse}$ (black circles) was calculated. The $RMSE_{Cal, full}$ (red upward triangles) was calculated based on the linear interpolation of all available offline samples (Figure S7D-F, filled and hollow symbols). The $RMSE_{Pred, full}$ (green downward triangles) was calculated for the prediction dataset and the respective offline values (Figure 4E-H, hollow symbols). For the calculation of the $RMSE_{Pred, full, -100\%}$ (blue diamonds), the cultures with 697.9 mg/L phosphate (Figure 4E-H, Figure S7D-F, purple circles) were excluded.

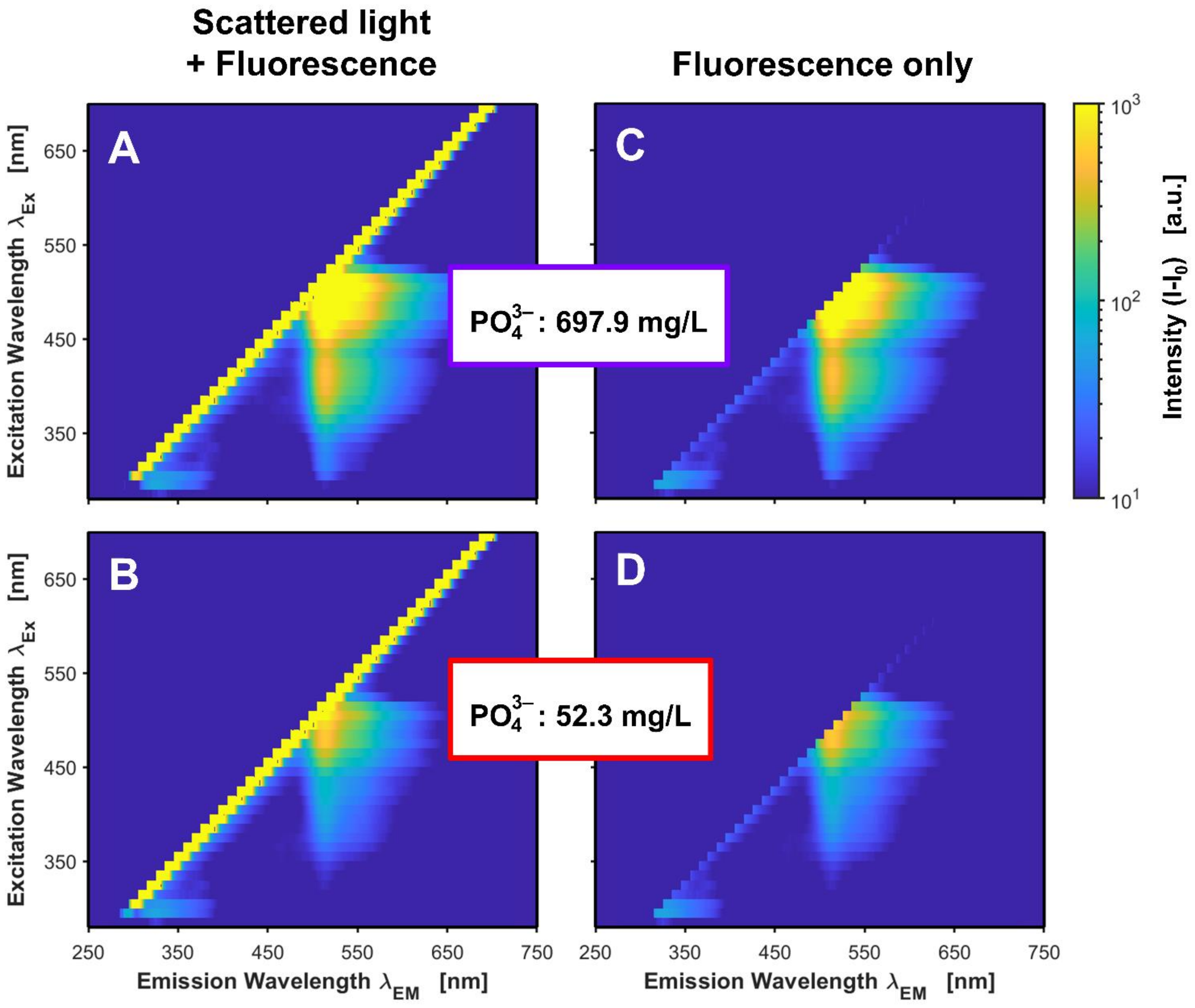

Figure S10: Exemplary 2D spectra of *H. polymorpha* RB11 pC9-*FMD* (P_{FMD}-GFP) cultures after 24 h of cultivation cultivated at initial phosphate (PO_4^{3-}) concentrations of (A, C) 697.9 mg/L and (B, D) 52.3 mg/L. Spectra (A, B) including scattered light and fluorescence, as well as (C, D) spectra including fluorescence only are shown. The scattered light exclusion was conducted in silico, as described in the material and methods section. Spectroscopic measurement settings: excitation wavelength range = 280 nm – 700 nm (step size = 10 nm), emission wavelength range = 278 nm – 720 nm (step size = 0.45 nm), integration time = 30 ms. Cultivation conditions: 48-well microtiter plate with round geometry, modified SYN6-MES medium, liquid volume = 800 µL, shaking diameter = 3 mm, shaking frequency = 1000 rpm, temperature = 30 °C.

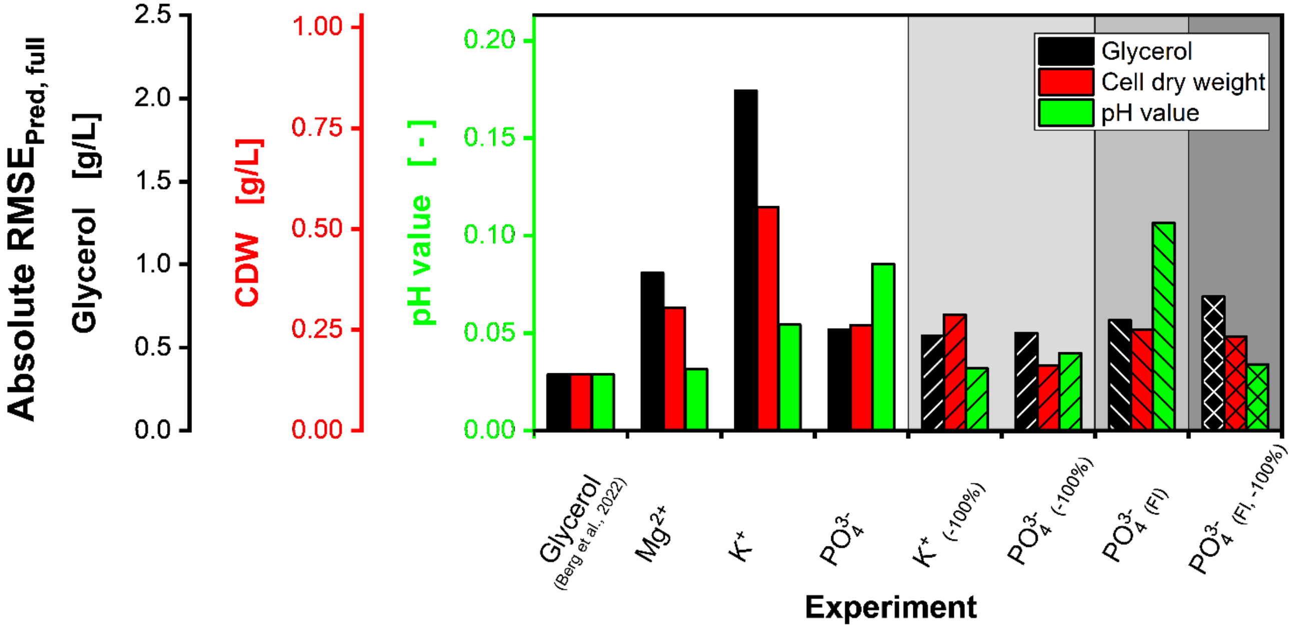

Figure S11: Comparison of absolute RMSE$_{Pred, full}$ for glycerol, CDW and pH value for the PLS models generated in Berg et al. [41] and this study. Plain columns show the RMSE based on the complete prediction datasets from this study and the study by Berg et al. [41]. Backward diagonal hatched columns describe the RMSE based on the complete prediction dataset, except the culture holding the initial concentration of the respective second substrate (-100%). Forward-hatched columns describe the RMSE calculated for spectral online datasets including fluorescence (Fl) only. For the diagonal cross-hatched columns, additionally, the cultures with the initial concentration of the respective second substrate, according to Jeude et al. [52], were excluded (-100%). Exact values as well as the number of LVs used for each model are summarized in Additional File 2, Table S1.

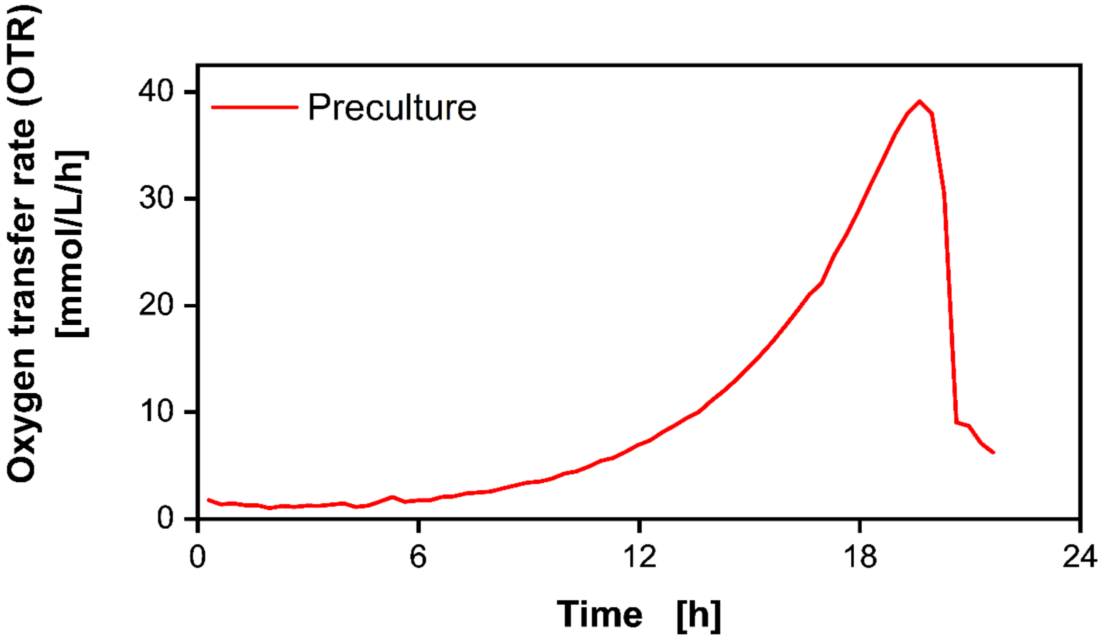

Figure S12: Exemplary Oxygen transfer rate (OTR) measured for the preculture of *Hansenula polymorpha* RB11 pC9-*FMD* (P_{FMD}-GFP). Cultivation conditions: modified SYN6-MES medium, 10 g/L glycerol, initial optical density = 0.1, 250 mL shake flask, filling volume = 10 mL, shaking frequency = 350 rpm, shaking diameter = 50 mm, T = 30°C

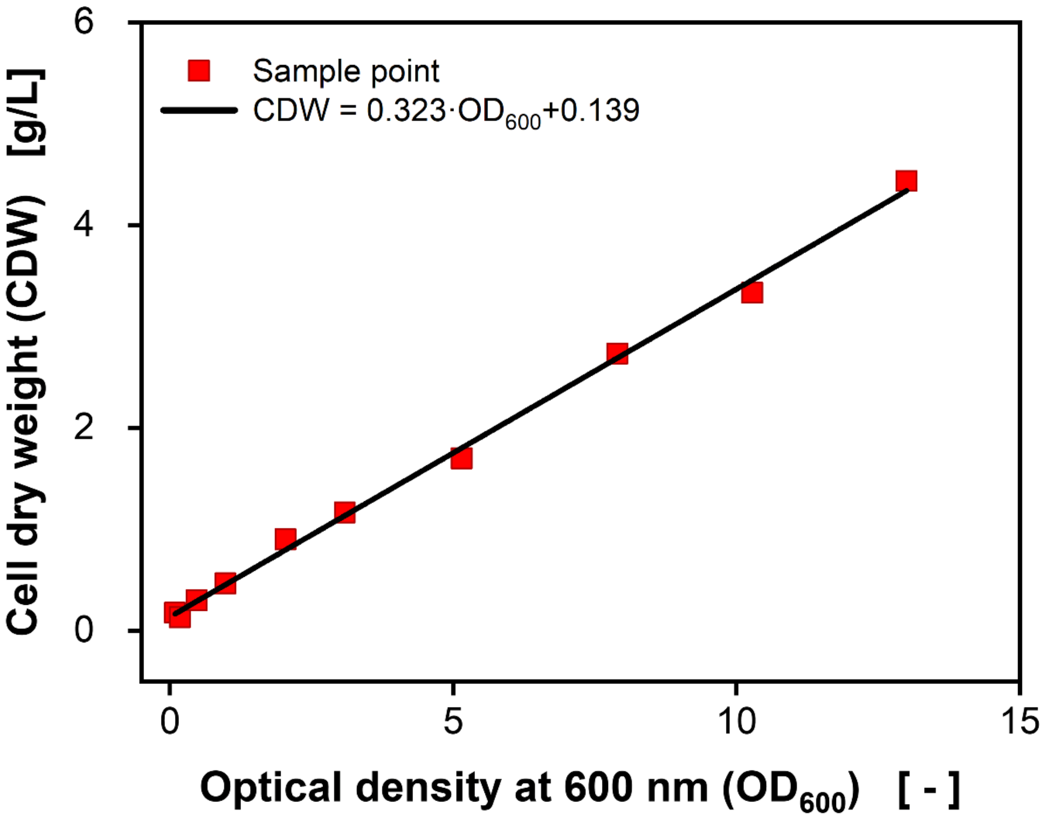

Figure S13: Linear correlation between cell dry weight (CDW) and optical density at 600 nm (OD_{600}). Cultures were grown in a modified SYN6-MES medium according to the preculture protocol. The shake flask cultures were harvested during exponential growth before the dilution series were prepared with fresh medium. For the OD_{600} measurement of the diluted series, additional dilution was conducted to allow measurements between 0.1 and 0.3. Adapted from Berg et al. [41].
