# Supplementary material for: Assessing the capabilities of 2D fluorescence monitoring in microtiter plates with data-driven modeling for secondary substrate limitation experiments of Hansenula polymorpha
Source: J Biol Eng. 2023 Feb 13;17:12. doi: 10.1186/s13036-023-00332-0 (PMC9926666; doi:10.1186/s13036-023-00332-0)
Supplement: Supplementary file 2 — Additional file 2: Table S1. Absolute and relative RMSE of calibration and prediction for PLS models of glycerol, CDW, and pH-value. Results are shown for the experiments of this study and the previous study for glycerol variation [41]. The RMSEs for calibration and prediction are shown for the shortest available sampling interval (RMSECal,full, RMSEPred,full). The relative RMSE, shown in brackets, is calculated based on the offline parameter range for the respective experiment. [file 13036_2023_332_MOESM2_ESM.pdf]

			Glycerol variation (Berg et al., 2022)	Magnesium limitation	Potassium limitation	Potassium limitation (-100%)	Phosphate limitation	Phosphate limitation (-100%)	Phosphate limitation (FI)	Phosphate limitation (FI, -100%)
Glycerol [g/L]		Range	0.0 – 9.0	0.0 – 11.68	0.0 – 10.93	0.0 – 10.93	0.0 – 10.86	0.0 – 10.86	0.0 – 10.86	0.0 – 10.86
	RMSE	Calibration	0.31 (3.5%) LV2	0.57 (5.1%) LV2	0.51 (4.7%) LV4		0.58 (5.2%) LV5		0.56 (4.9%) LV5	
		Prediction	0.34 (3.8%)	0.95 (8.5%)	2.05 (18.7%)	0.57 (5.2%)	0.61 (5.6%)	0.59 (5.4%)	0.67 (6.1%)	0.81 (7.4%)
CDW [g/L]		Range	0.0 – 3.67	0.0 – 4.67	0.0 – 3.79	0.0 – 3.42	0.0 – 3.95	0.0 – 3.82	0.0 – 3.95	0.0 – 3.82
	RMSE	Calibration	0.12 (3.2%) LV2	0.18 (3.8%) LV3	0.15 (4.0%) LV3		0.12 (3.0%) LV5		0.13 (3.3%) LV5	
		Prediction	0.14 (3.8%)	0.30 (6.5%)	0.55 (14.6%)	0.29 (8.4%)	0.26 (6.6%)	0.16 (4.3%)	0.25 (6.3%)	0.23 (6.2%)
pH value [-]		Range	6.0 – 5.5	5.45 – 5.98	5.19 – 5.98	5.49 – 5.98	5.18 – 6.18	5.44 – 6.18	5.18 – 6.18	5.44 – 6.18
	RMSE	Calibration	0.023 (4.3%) LV2	0.025 (4.9%) LV2	0.034 (4.3%) LV3		0.038 (3.8%) LV3		0.040 (4.0%) LV3	
		Prediction	0.029 (5.3%)	0.032 (6.0%)	0.054 (6.9%)	0.032 (6.5%)	0.085 (8.5%)	0.040 (5.4%)	0.106 (10.6%)	0.034 (4.6%)

Table S1: Absolute and relative RMSE of calibration and prediction for PLS models of glycerol, CDW, and pH-value. Results are shown for the experiments of this study and the previous study for glycerol variation [41] The RMSEs for calibration and prediction are shown for the shortest available sampling interval ($RMSE_{Cal,full}$, $RMSE_{Pred,full}$). The relative RMSE, shown in brackets, is calculated based on the offline parameter range for the respective experiment.
